# A Dual-Mechanism Enhanced Secretary Bird Optimization Algorithm and Its Application in Engineering Optimization

**DOI:** 10.3390/biomimetics10100679

**Published:** 2025-10-09

**Authors:** Changzu Chen, Li Cao, Binhe Chen, Yaodan Chen, Xinxue Wu

**Affiliations:** 1 School of Electronics and Electrical Engineering, Wenzhou University of Technology, Wenzhou 325035, China; chenchangzu@wzut.edu.cn (C.C.); caoli198723@163.com (L.C.); 20210378@wzut.edu.cn (B.C.); chenyaodan@wzut.edu.cn (Y.C.); 2 Wenzhou Branch Data Research Center, China Tower Corporation Limited, Wenzhou 325000, China

**Keywords:** secretary bird optimization algorithm, swarm intelligence, optimal neighborhood perturbation, reverse learning strategy, engineering optimization

## Abstract

The secretary bird optimization algorithm is a recently developed swarm intelligence method with potential for solving nonlinear and complex optimization problems. However, its performance is constrained by limited global exploration and insufficient local exploitation. To address these issues, an enhanced variant, ORSBOA, is proposed by integrating an optimal neighborhood perturbation mechanism with a reverse learning strategy. The algorithm is evaluated on the CEC2019 and CEC2022 benchmark suites as well as four classical engineering design problems. Experimental results demonstrate that ORSBOA achieves faster convergence, stronger robustness, and higher solution quality than nine state-of-the-art algorithms. Statistical analyses further confirm the significance of these improvements, validating the effectiveness and applicability of ORSBOA in solving complex optimization tasks.

## 1. Introduction

The growing complexity of engineering optimization, image recognition, intelligent manufacturing, and network scheduling has brought high-dimensional, multimodal, and strongly constrained problems to the forefront of intelligent optimization research [1,2,3]. Deterministic methods often struggle with nonlinear, non-differentiable, or black-box objectives, whereas metaheuristics have become mainstream tools due to their strong global search ability, problem-independence, and simple structures [4,5,6,7]. Among them, nature-inspired algorithms—such as particle swarm optimization (PSO) [8], gray wolf optimization (GWO) [9], whale optimization (WOA) [10], and beetle antenna search (BAS) [11]—have been widely applied and rigorously studied.

The secretary bird optimization algorithm (SBOA) is a relatively new swarm intelligence method inspired by the predatory behavior of African secretary birds [12]. It models vision-guided tracking and multi-stage sprint attacks to balance global exploration and local exploitation. Prior studies report promising global search capability and rapid convergence on diverse benchmarks [13,14,15,16]. Nevertheless, SBOA still exhibits limitations, including insufficient local exploitation, difficulty in maintaining population diversity, and a tendency to be trapped in local optima—particularly in high dimensions [17]. In addition, its parameter-tuning mechanism is relatively static, reducing adaptability across scenarios [18].

To alleviate these issues, several improved variants have been proposed. Mai et al. [19] introduced CSBOA by combining chaotic initialization with improved differential mutation and crossover, achieving faster convergence and better solution quality on CEC2017/CEC2022 and engineering problems. Yan et al. [20] incorporated a hybrid chaotic mutation strategy to enhance SBOA, while Zhu et al. [21] proposed QHSBOA, which integrates quantum computing concepts and dynamic boundary adjustment for classification tasks. Although these variants deliver partial gains, many trade off exploration against exploitation (or vice versa) and thus fail to offer a balanced performance across heterogeneous landscapes.

In this work, the Optimal Neighborhood Perturbation and Reverse Learning Strategy based Secretary Bird Optimization Algorithm (ORSBOA) is proposed. Unlike prior improvements, ORSBOA simultaneously strengthens exploration and exploitation through two complementary mechanisms: (i) optimal neighborhood perturbation (ONP) [22], which refines local search by perturbing around the current best solutions, and (ii) a reverse learning strategy (RLS) [23], which enriches global exploration by generating candidate solutions in regions opposite to the current population. Together, these mechanisms enhance convergence speed, stability, and robustness [24,25,26,27,28].

The performance of ORSBOA is assessed on the CEC2019 [29] and CEC2022 [30] benchmark suites and four classical engineering design problems: welded beam, three-bar truss, cantilever beam, and compression spring [31,32]. Comparisons against nine mainstream algorithms evaluate accuracy, stability, and robustness; statistical tests and visualization analyses substantiate the significance and effectiveness of the observed improvements.

The main contributions are threefold:

- An improved algorithm (ORSBOA) is proposed that integrates ONP and RLS to concurrently enhance exploration and exploitation.
- The effectiveness of ORSBOA is systematically validated on benchmark suites and engineering problems, demonstrating superior accuracy, stability, and efficiency.
- Extensive experiments and statistical analyses confirm the superiority and adaptability of ORSBOA, offering practical value and methodological insight for the design of swarm intelligence algorithms.

## 2. Dual-Mechanism Enhanced Secretary Bird Optimization Algorithm

SBOA simulates the predatory behavior of secretary birds hunting snakes by mapping vision-guided tracking and multi-stage strikes into a position-update model for numerical optimization. The population explores the search space through stochastic position updates, where randomness regulates the effective exploration radius. However, on high-dimensional and multimodal problems, SBOA is prone to premature convergence due to limited local exploitation and a largely unidirectional search bias, which can yield unstable performance.

To overcome these limitations, two complementary mechanisms are embedded into the SBOA framework, forming ORSBOA: (i) optimal neighborhood perturbation, which intensifies local search by applying controlled perturbations around elite solutions to refine promising regions while preserving diversity; and (ii) a reverse learning strategy, which strengthens global exploration by generating candidate solutions in regions opposite to the current population distribution. Integrating ONP and RLS within a unified search scheme yields a more favorable exploration–exploitation balance and improves convergence speed, stability, and robustness on complex landscapes.

### 2.1. Secretary Bird Optimization Algorithm

SBOA models two characteristic behaviors of the secretary bird—wide-area search and precision strike—which correspond to the algorithm’s exploration and exploitation phases. The overall mechanism follows three sequential stages: initialization, exploration, and exploitation. In the initialization stage, a population is generated within the search bounds and the best individual is identified. During exploration, stochastic motions with random directions and adaptive step sizes broaden search coverage and maintain diversity. In the exploitation stage, the search becomes more directed, refining solutions around elite individuals with smaller steps to improve quality. By alternating exploration and exploitation, SBOA aims to balance global coverage and local refinement, and the detailed procedures are described as follows:

(1) Initialization

SBOA begins with population initialization. In a D-dimensional search space, an initial population consisting of N individuals is generated. Each individual $X_{i}$ represents a potential solution to the problem, and its initial position is randomly generated using the following equation:

(1) $$X_{i}^{0}=lb+\mathrm{rand}(0,1)\cdot (ub-lb)$$

where lb and ub represent the lower and upper bound vectors of the search space, respectively, and rand(0,1) is a uniformly distributed random number within the interval (0,1).

(2) Exploration phase

In the exploration phase, SBOA simulates the secretary bird’s behavior of locating, exhausting, and attacking prey through a three-stage search process. The entire predation activity is divided into three equal time intervals, corresponding to the three phases of predation: searching for prey, exhausting prey, and attacking prey.

In the searching for prey stage, inspired by the idea of differential evolution, the position update of individuals is governed by the following equation:

(2) $$\left\{\begin{array}{l} \mathrm{While}\;t<\frac{1}{3}T,\\ X_{i}^{\mathrm{new},P1}=X_{i}+(X_{\mathrm{random}1}-X_{\mathrm{random}2})\cdot R_{1},\\ X_{i}=\left\{\begin{array}{ll} X_{i}^{\mathrm{new},P1}, & \mathrm{if}\;F_{i}^{\mathrm{new},P1}<F_{i},\\ X_{i}, & \mathrm{else} \end{array}\right. \end{array}\right.$$

where t is the current iteration number, T is the maximum number of iterations, $X_{i}^{\mathrm{new},P1}$ denotes the new state of the i-th secretary bird in the first stage, $X_{i}$ is the current position of the i-th secretary bird, $X_{\mathrm{random}1}$ and $X_{\mathrm{random}2}$ are randomly selected candidate solutions in the first-stage iteration, $R_{1}$ is a randomly generated number in the interval [0,1], and $F_{i}^{\mathrm{new},P1}$ represents the fitness value of the objective function.

In the exhausting prey stage, Brownian motion [33,34] is introduced to simulate the random movement of the secretary bird, thereby increasing the stochasticity of the search. The position update in the attacking prey stage is given by the following:

(3) $$\left\{\begin{array}{l} RB=\mathrm{randn}(1,\mathrm{Dim}),\\ \mathrm{While}\;\frac{1}{3}T<t<\frac{2}{3}T,\\ X_{i}^{\mathrm{new},P1}=x_{\mathrm{best}}+\exp\left({\left(\frac{t}{T}\right)}^{\gamma }\right)\times (RB-0.5)\times (x_{\mathrm{best}}-X_{i}),\\ X_{i}=\left\{\begin{array}{ll} X_{i}^{\mathrm{new},P1}, & \mathrm{if}\;F_{i}^{\mathrm{new},P1}<F_{i},\\ X_{i}, & \mathrm{else} \end{array}\right. \end{array}\right.$$

where RB is a random number generated from a standard normal distribution, and $x_{\mathrm{best}}$ denotes the current best solution.

In the attacking prey stage, the current best solution $X_{\mathrm{best}}$ is used to guide the search, and the position update of individuals is defined as follows:

(4) $$\left\{\begin{array}{l} \mathrm{While}\;t>\frac{2}{3}T,\\ X_{i}^{\mathrm{new},P1}=x_{\mathrm{best}}+\left(\left(1-\frac{t}{T}\right)\cdot \left(\frac{2t}{T}\right)\right)\times X_{i}\times RL,\\ X_{i}=\left\{\begin{array}{ll} X_{i}^{\mathrm{new},P1}, & \mathrm{if}\;F_{i}^{\mathrm{new},P1}<F_{i},\\ X_{i}, & \mathrm{else} \end{array}\right. \end{array}\right.$$

where RL is a random number in the interval (0,0.5).

(3) Exploitation phase

In the exploitation phase, SBOA simulates the secretary bird’s camouflage and escape strategies. When the secretary bird senses a threat, it may choose either to camouflage itself or to flee rapidly. In the algorithm, the decision to perform camouflage (local fine-tuning) or escape (global search) is made based on the difference between the fitness value of the current solution and that of the historical best solution. $C_{1}$ corresponds to the camouflage phase, while $C_{2}$ represents the escape phase. The position update of individuals is given by the following:

(5) $$\begin{array}{l} \left\{\begin{array}{l} \mathrm{if}\;\mathrm{rand}<r_{i}\;C_{1}:\\ X_{i}^{\mathrm{new},P2}=x_{\mathrm{best}}+(2RB-1)\left(1-\frac{t}{T}\right)\times X_{i},\\ \mathrm{else}\;C_{2}:\\ X_{i}^{\mathrm{new},P2}=X_{i}+R_{2}\times \left(X_{\mathrm{random}}-K\times X_{i}\right),\\ X_{i}=\left\{\begin{array}{ll} X_{i}^{\mathrm{new},P2}, & F_{i}^{\mathrm{new},P2}<F_{i},\\ X_{i}, & \mathrm{else} \end{array}\right. \end{array}\right.\\ K=\mathrm{round}(1+\mathrm{rand}(1,1)) \end{array}$$

Despite its novelty, SBOA exhibits several practical limitations. Its fixed perturbation scheme and largely unidirectional updates can erode population diversity; in high-dimensional, complex landscapes this often precipitates premature convergence to local optima and weakens global exploration [13]. Moreover, the local-search step size lacks adaptive regulation: overly large steps overshoot promising basins and destabilize convergence, whereas overly small steps slow progress and increase the risk of entrapment in local minima. Collectively, these issues degrade solution quality, reduce robustness, and constrain SBOA’s effectiveness on challenging multimodal and constrained problems.

### 2.2. Optimal Neighborhood Perturbation

Neighborhood-based search has proven effective across metaheuristics—for example, local perturbations in PSO to escape local optima, neighborhood-driven mutations in differential evolution to sharpen solutions, and perturbation moves in artificial bee colony to enhance diversity and exploitation. These studies consistently show that neighborhood perturbation can improve convergence accuracy and robustness.

In ORSBOA, ONP is embedded into the SBOA framework to strengthen local exploitation, while RLS preserves broad exploration, yielding a more balanced search process. ONP operates by generating small, controlled perturbations within a bounded neighborhood of the current best solution, thereby directing fine-grained refinement toward high-quality regions of the search space. By focusing computation around promising areas without sacrificing population diversity, ONP reduces premature convergence and stabilizes performance on high-dimensional, multimodal problems—complementing RLS to maintain global coverage.

Concretely, ONP perturbs the current global best within a dynamic neighborhood whose radius shrinks over time, enabling a coarse-to-fine local search and mitigating premature convergence on high-dimensional, multimodal landscapes. Specifically, a dynamic neighborhood $N(X_{\mathrm{best}},r_{t})$ is constructed based on the current global best solution $X_{\mathrm{best}}$, where $r_{t}$ decays exponentially with the increase in the number of iterations t. The calculation formula is given by the following:

(6) $$r_{t}=r_{0}\cdot \exp(-k\cdot t/T)$$

where $r_{0}$ is the initial neighborhood radius, k is the decay control parameter, and T is the maximum number of iterations. In the early stages of the algorithm, a larger $r_{t}$ enables broader neighborhood exploration, which facilitates the discovery of new potential solutions. As the iterations progress, $r_{t}$ gradually decreases, concentrating the search on the local neighborhood of the optimal solution for fine-tuned exploitation, thereby improving the solution quality.

A hybrid perturbation operator combining Gaussian perturbation and Cauchy perturbation is designed, and the perturbation mode is dynamically selected based on a probability p. When rand(0,1)<p, Gaussian perturbation is used to perform fine-grained search within the neighborhood. The corresponding formula is as follows:

(7) $$X_{\mathrm{new}}^{1}=X_{\mathrm{best}}+\alpha \cdot \mathrm{Gauss}(0,1)$$

where α is the parameter controlling the intensity of the Gaussian perturbation, and Gauss(0,1) is a random number following a standard Gaussian distribution. When rand(0,1)≥p, Cauchy perturbation is employed to escape from local optima. The update formula is as follows:

(8) $$X_{\mathrm{new}}^{2}=X_{\mathrm{best}}+\beta \cdot \mathrm{Cauchy}(0,1)$$

where β is the parameter controlling the intensity of the Cauchy perturbation, and Cauchy(0,1) is a random number following a standard Cauchy distribution.

The local optimum escape mechanism of this strategy is analyzed using a Markov chain model. Suppose the algorithm becomes trapped at a local optimum $X^{*}$. In the standard SBOA, the probability of escaping the local optimum in each iteration is denoted as $P_{1}$.

(9) $$P_{1}=O\left(1/T\right)$$

Under the ONP strategy, due to the introduction of a dynamic neighborhood and the hybrid perturbation operator, the probability of escaping the local optimum in each iteration increases to $P_{2}$.

(10) $$P_{2}=O(1/\sqrt{T})$$

This indicates that the ONP strategy can significantly enhance the algorithm’s ability to escape local optima and improve its global convergence performance.

### 2.3. Reverse Learning Strategy

RLS is a widely used diversity–enhancement mechanism in swarm intelligence. For a given solution, an opposite candidate is generated by reflecting the point with respect to the variable bounds. The intuition is that when a solution lies far from the global optimum, its opposite has a higher chance of being closer. Injecting these opposite candidates increases population diversity, mitigates premature convergence, and strengthens global exploration.

In ORSBOA, RLS complements ONP by supplying exploration-oriented candidates while ONP concentrates on local refinement. Opposite solutions are generated dynamically and integrated selectively: candidates compete with their counterparts (or with low-quality individuals), and replacements occur only when fitness is improved or when diversity falls below a threshold. This selective integration enriches information without disrupting convergence.

For the current solution $X_{i}$, a dynamic opposite solution $X_{i}^{\mathrm{ob}}$ is generated using the following formula:

(11) $$X_{i}^{\mathrm{ob}}=lb+ub-X_{i}$$

where r is a random number uniformly distributed within the interval [0,1]. A fitness-weighted factor $w_{i}$ is introduced and calculated based on the fitness value $f(X_{i})$ of the current solution $X_{i}$ and the average fitness value $f_{\mathrm{avg}}$ of the population, as given by the following formula:

(12) $$w_{i}=\exp(-f(X_{i})/f_{\mathrm{avg}})$$

The weighted factor $w_{i}$ is used to achieve adaptive integration of the current solution and its opposite solution, as defined by the following formula:

(13) $$X_{i}^{\mathrm{new}}=w_{i}\cdot X_{i}+(1-w_{i})\cdot X_{i}^{\mathrm{ob}}$$

When $f(X_{i})$ is relatively small, $w_{i}$ becomes large, indicating that the current solution is of higher quality; thus, the fused solution tends to retain more characteristics of the current solution. Conversely, when $f(X_{i})$ is relatively large, $w_{i}$ becomes small, and the fused solution is more inclined to incorporate information from the opposite solution, thereby introducing diversity while preserving desirable features of high-quality solutions.

### 2.4. Optimal Neighborhood Perturbation and Reverse Learning Strategy Based Secretary Bird Optimization Algorithm

Building on SBOA, ORSBOA integrates ONP and RLS to enhance search capability and stability across different phases. The method establishes a unified hybrid framework that tightly couples global exploration with local exploitation: RLS injects exploration-oriented candidates from opposite regions of the search space, while ONP conducts fine-grained refinement around elite solutions. The two mechanisms operate in a mutually reinforcing, dynamically balanced manner to reduce premature convergence and improve convergence reliability on complex landscapes. The ORSBOA framework contains the following five main modules:

(1) Initialization

At the beginning of the search, the algorithm generates an initial population uniformly at random within the solution space $\left[X_{\min},X_{\max}\right]$, based on the predefined population size N and problem dimension D. Each individual $X_{i}$ represents a potential solution. After population initialization, fitness evaluation is performed, and the current best solution $X_{\mathrm{best}}$ is recorded.

(2) Main search mechanism of the SBOA

In each generation, ORSBOA retains the core update model from SBOA, which simulates the secretary bird’s attack behavior. Individuals are guided to leap toward the current global best solution $X_{\mathrm{best}}$, while random perturbations are added to enhance the stochastic nature of the search. The update equation is as follows:

(14) $$X_{i}^{t+1}=X_{i}^{t}+\alpha \cdot (X_{\mathrm{best}}^{t}-X_{i}^{t})+\beta \cdot N(0,1)\cdot (ub-lb)$$

where α and β are the control factors for the search direction and perturbation intensity, respectively, and N(0,1) denotes a standard normal distribution. This strategy helps maintain directional search toward potential optimal regions of the solution space.

(3) Optimal neighborhood perturbation

To enhance the local search capability of elite individuals, a perturbation operation is applied after each iteration with a certain probability $P_{perturb}$. The operation targets either the current global best individual or an elite individual. The perturbation region is constructed around the global best solution, and several perturbed individuals are generated using the following equation:

(15) $$X_{perturb}=X_{\mathrm{best}}+\delta_{t}\cdot N(0,1)$$

where $\delta_{t}$ is the scaling factor that controls the intensity of the perturbation. If the fitness of the perturbed individual is better than that of the original best solution, the global best position is updated accordingly; otherwise, the original state is retained. This mechanism is designed to improve the precision of local exploitation and enhance the depth of local solution refinement.

(4) Reverse learning strategy

To maintain population diversity and prevent premature convergence, ORSBOA applies a perturbation operation to the current global best or elite individuals in each generation with a probability $P_{ob}$. For the current solution $X_{i}$, a dynamic opposite solution $X_{i}^{\mathrm{ob}}$ is generated using the following equation:

(16) $$X_{i}^{\mathrm{ob}}=lb+ub-r\cdot X_{i}$$

where r is a random number uniformly distributed in the interval [0,1]. Unlike traditional opposition-based learning strategies, the dynamic opposition-based learning strategy introduces a random number r, which breaks the fixed mapping relationship of standard opposition and increases the randomness of the solution space. This allows the opposite solution to more broadly cover the search space. If the opposite solution is better than the original one, it replaces the current individual. This operation expands the coverage of the solution space and enhances the information gain of the population.

(5) Termination and convergence output

The iteration process terminates when the maximum number of iterations T is reached, or when there is no significant improvement in the global best fitness value over several consecutive generations. At that point, the current global best individual and its corresponding objective function value are returned as the output.
**Algorithm 1 Pseudo code of ORSBOA**Inputs: the maximum number of iterations is *T*, the size of the population is *N*. Output: optimal position $X_{\mathrm{best}}$ and fitness value $f(X_{\mathrm{best}})$.Initialize the population according to Equation (1) and specify the relevant parameters;$\mathrm{Evaluate}\;\mathrm{fitness}\;f(X_{i})$$\;\mathrm{and}\;\mathrm{determine}\;\mathrm{the}\;\mathrm{best}\;\mathrm{solution}\;X_{\mathrm{best}}$For t=1 to T do   ----- Exploration Phase -----   $a.\;\mathrm{For}\;\mathrm{each}\;\mathrm{individual}\;X_{\mathrm{best}}$:        If t<T/3:           $\mathrm{Update}\;X_{i}$ according to Equation (2)        Else if t<2T/3:           $\mathrm{Update}\;X_{i}$ according to Equation (3)        Else:           $\mathrm{Update}\;X_{i}$ according to Equation (4)        End if        $\mathrm{Update}\;X_{i}$   ----- Exploitation Phase -----   $b.\;\mathrm{For}\;\mathrm{each}\;X_{i}$:        If rand>0.5:           $\mathrm{Update}\;X_{i}$$\;\mathrm{according}\;\mathrm{to}\;C_{1}$ in Equation (5)        Else:           $\mathrm{Update}\;X_{i}$$\;\mathrm{according}\;\mathrm{to}\;C_{2}$ in Equation (5)        End if           $\mathrm{Update}\;X_{i}$$\;\mathrm{and}\;X_{best}$   ----- Global Update -----        c. Perform global update according to Equations (13) and (14)        $d.\;\mathrm{If}\;\mathrm{new}\;\mathrm{position}\;\mathrm{is}\;\mathrm{better},\;\mathrm{update}\;X_{i}$$\;\mathrm{and}\;X_{best}$End For$\mathrm{Output}\;\mathrm{the}\;\mathrm{best}\;\mathrm{solution}\;X_{best}$

**Figure 1:**
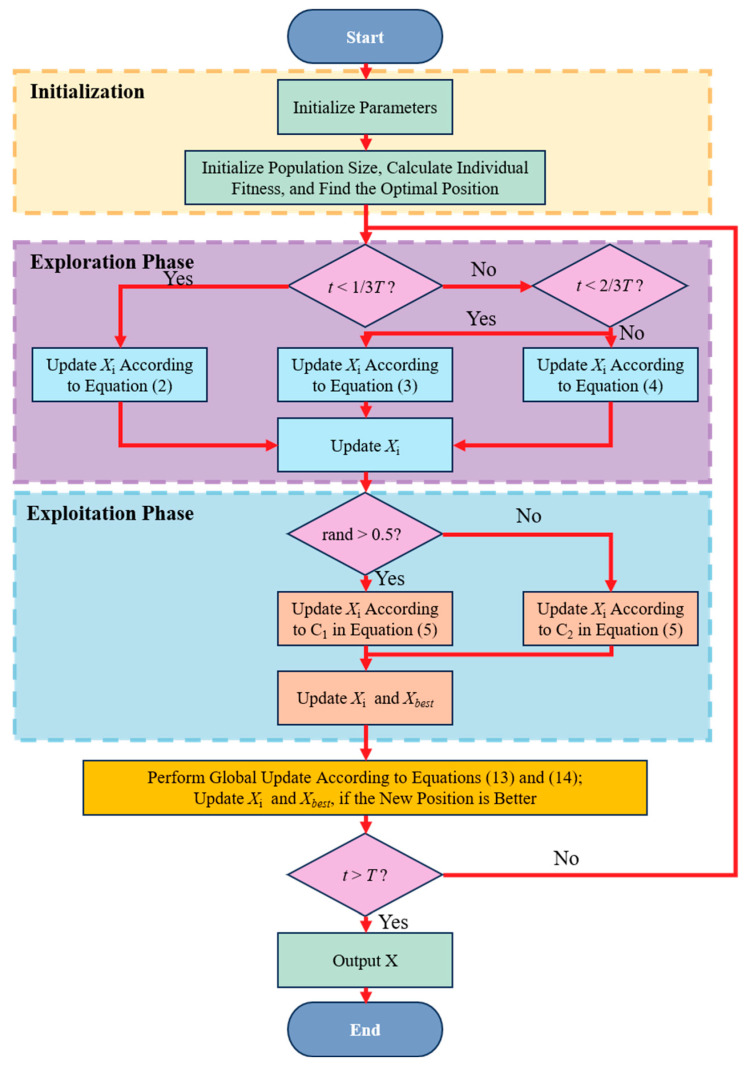
Flowchart of the ORSBOA.

The whole hybrid search process is shown in Figure 1 and detailed in *Algorithm 1 (Pseudo code of ORSBOA)*. ORSBOA maintains the strong global search capability, and at the same time, it constructs multi-level search dynamics through the dual enhancement mechanism. While maintaining the strong global search capability, ORSBOA constructs multi-level search dynamics through the dual enhancement mechanism, which significantly improves the adaptability of the algorithm to the complex functional landscape and enhances the convergence performance.

Within the optimization cycle, RLS and ONP play complementary roles: RLS injects opposite candidates to enlarge coverage and sustain diversity, whereas ONP performs controlled, neighborhood-based refinements around promising individuals. Their coordinated use enables a dynamic exploration–exploitation balance, yielding greater stability and robustness than baseline SBOA.

### 2.5. Comparison with Related Hybrid Approaches

Hybrid schemes that couple OBL with neighborhood perturbation have been extensively explored in metaheuristics. Many variants diversify the search via OBL while embedding neighborhood-based moves to improve convergence accuracy [35,36]. However, their triggers and intensities are often static, which limits adaptability across heterogeneous problem landscapes.

By contrast, ORSBOA integrates ONP and RLS within a dynamically coordinated framework. RLS injects opposite candidates to expand coverage and sustain diversity, whereas ONP applies controlled, bounded refinements around elite solutions. The two operators are scheduled and selected adaptively, yielding an on-the-fly balance between exploration and exploitation and reducing susceptibility to premature convergence.

Empirical comparisons on benchmark suites show that ORSBOA attains faster convergence and higher solution accuracy than closely related OBL–local-search hybrids [37]. In particular, RLS enhances global exploration—especially early or under stagnation—while ONP strengthens late-stage refinement without incurring notable computational overhead. This co-designed interaction distinguishes ORSBOA from prior hybrids that apply opposition or local search independently, delivering emergent gains absent in static couplings [38].

The computational complexity of ORSBOA remains O(N⋅D⋅T),where N is population size, D the dimensionality, and T the iteration budget. The added costs of RLS and ONP are linear in N and D and thus do not alter the overall order relative to baseline SBOA, preserving practical scalability to medium- and large-scale problems.

## 3. Experimental Comparison and Result Analysis

To comprehensively assess ORSBOA, two widely used benchmark suites are employed: CEC2019 and CEC2022, both originating from the IEEE CEC single-objective optimization competitions and serving as de facto standards in evolutionary computation. CEC2019 includes unimodal, multimodal, hybrid, and composition functions with scalable dimensionality, primarily testing an algorithm’s ability to balance exploration and exploitation. CEC2022 features more rugged landscapes with multiple local optima, hybrid structures, and strong inter-variable dependencies, providing a stricter test of robustness and adaptability. Using both suites offers complementary perspectives on effectiveness across heterogeneous problem characteristics and difficulty levels.

### 3.1. Experimental Setup

All experiments were conducted in MATLAB R2021b on Windows 10 with an Intel Core i7-11800H CPU (Intel Corporation, Santa Clara, CA, USA; purchased in China) and 16 GB RAM. For reproducibility and fairness, all methods used identical budgets: population size N=30, maximum iterations $T_{\max}=1000$, and 30 independent runs per function. The problem dimension was set to D=10; variable bounds followed each suite’s specifications.

Comparative baselines comprise nine representative population-based optimizers: GWO [9], WOA [10], Harris Hawks optimization algorithm (HHO) [39], BAS [11], coyote optimization algorithm (COA) [40], fox optimization algorithm (FOX) [41], dung beetle optimization algorithm (DBO) [42], osprey optimization algorithm (OOA) [43] and SBOA. Control parameters for these algorithms were taken from their original sources or widely accepted recommendations; no method was retuned for this study. Uniform population sizes and iteration limits were applied across all algorithms to ensure a fair comparison.

Performance was summarized using Min (minimum), Std (standard deviation), Avg (arithmetic mean), Median, Worst, and Avg_time (average runtime). Statistical significance was examined via Wilcoxon rank-sum and Wilcoxon signed-rank tests [44,45]. Mean-rank diagrams and radar plots were used to visualize overall performance.

### 3.2. CEC2019 Experimental Results

In order to comprehensively evaluate the optimization performance of the proposed ORSBOA on standard benchmark functions, 10 functions (F1~F10) from the CEC2019 test set are selected as the benchmark test set. These functions cover single-peak, multi-peak, composite and complex hybrid types, and are characterized by non-trivial gradient, high-dimensional nonlinearity and dense local optimization. These features can comprehensively test the algorithm’s global search capability, convergence speed and stability under different problem complexities.

**Figure 2:**
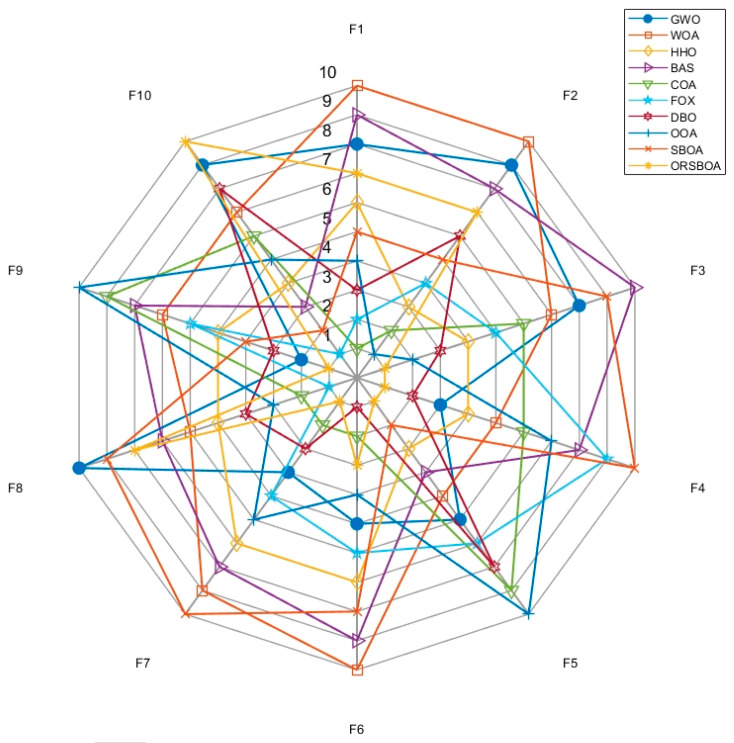
Radar diagram of ORSBOA and comparison algorithms on CEC2019.

Overall, ORSBOA performs well on most of the tested functions. As shown in the average ranked radar plots of the algorithms on F1~F10 in Figure 2, ORSBOA is consistently located in the center region of the radar plots for many functions, indicating its excellent overall performance. In contrast, traditional algorithms such as WOA, BAS, and SBOA appear more frequently at the outer edges of the radar plot, indicating their relatively weak stability and search efficiency.

**Figure 3:**
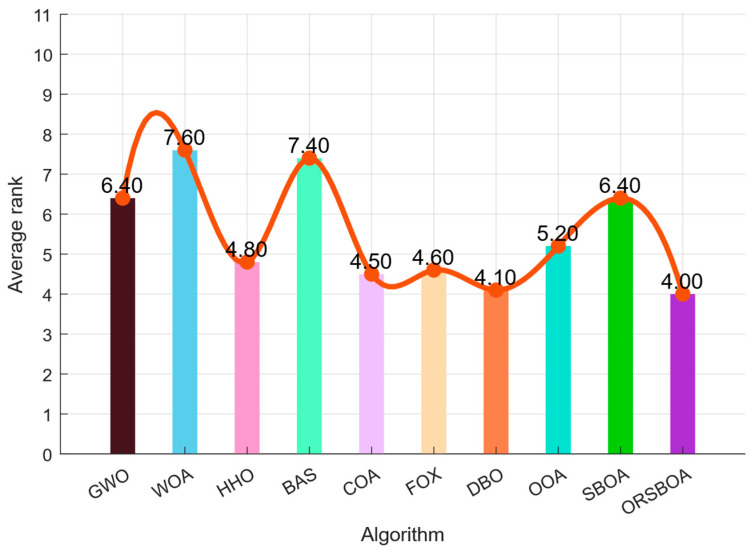
Plot of average ranking of ORSBOA and comparison algorithms on CEC2019.

Figure 3 shows the histogram of the average rankings of the 10 functions. ORSBOA achieves the best average ranking of 4.00, outperforming DBO (4.10), FOX (4.60), and COA (4.50), and significantly outperforming WOA (7.60) and BAS (7.40). These results clearly demonstrate the performance advantages of ORSBOA in solving complex optimization problems.

According to the numerical statistics shown in Table 1, ORSBOA performs well in key metrics such as the optimal, mean and median values. For example, in function F5, ORSBOA not only achieves single-digit mean fitness values, but also has a significantly lower standard deviation compared with other algorithms, indicating that it has a stronger ability to jump out of the local optimums in dense clustering regions. In most functions, ORSBOA’s solution fluctuates less and its worst-case values and standard deviation remain low, reflecting its excellent robustness and convergence stability.

Although its computation time is slightly higher than that of lightweight algorithms such as GWO and BAS, it is still within a reasonable range. Considering the solution accuracy and efficiency, ORSBOA has strong practical applicability to engineering problems.

In terms of statistical significance, the test results shown in Table 2 and Table 3 indicate that ORSBOA is significantly different from the conventional algorithm on most of the tested functions. This confirms the statistically significant advantage of the improvements introduced by ORSBOA. ORSBOA achieved statistically significant advantages on 9 out of 10 functions compared to SBOA, further validating the effectiveness of the optimal neighborhood perturbation and the dyadic learning-based strategy in enhancing the algorithm’s convergence paths and solution space exploration.

**Figure 4:**
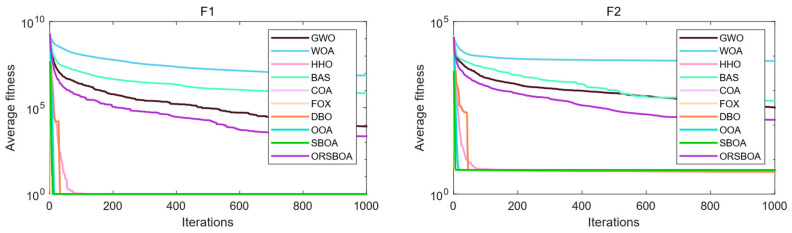

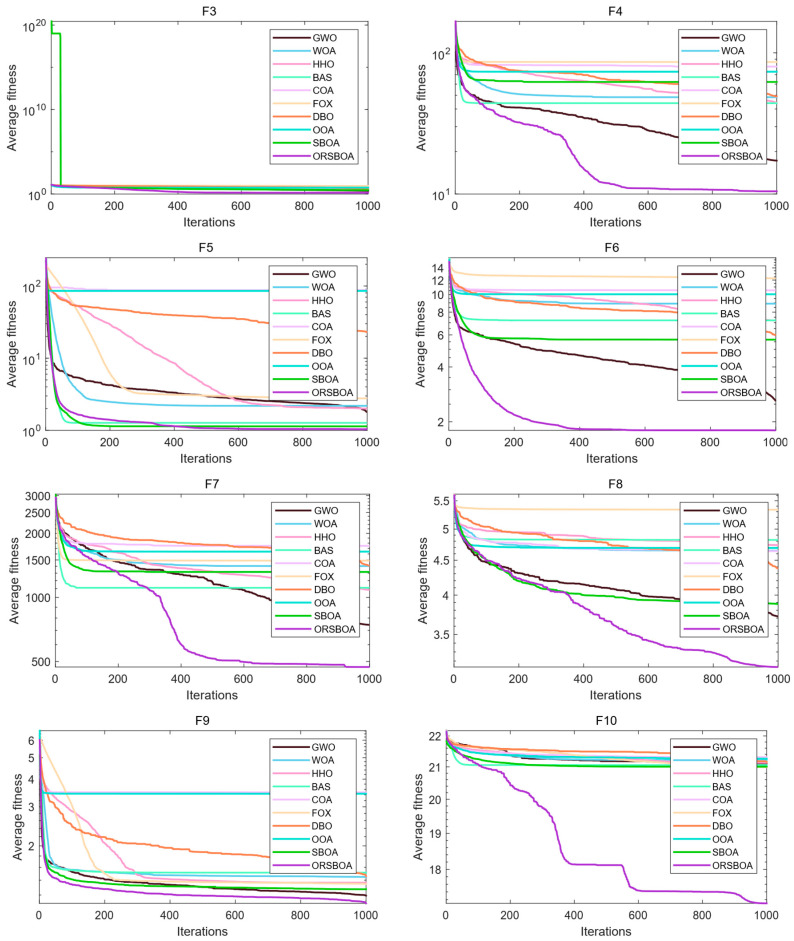
Average convergence curves of ORSBOA and comparison algorithms on CEC2019.

Figure 4 shows the average convergence curves of each algorithm on functions F1~F10, which are used to evaluate the convergence speed and the exploration ability of the algorithms in the later stages. From the trend, ORSBOA shows a faster decrease in adaptation in the early stages of multiple functions, reflecting a strong global exploration ability. In the middle and late stages, its convergence curve stabilizes, reflecting a strong local exploration ability. For example, in functions F5, F6 and F7, ORSBOA converges significantly faster than the other algorithms and finally achieves better fitness values.

**Figure 5:**
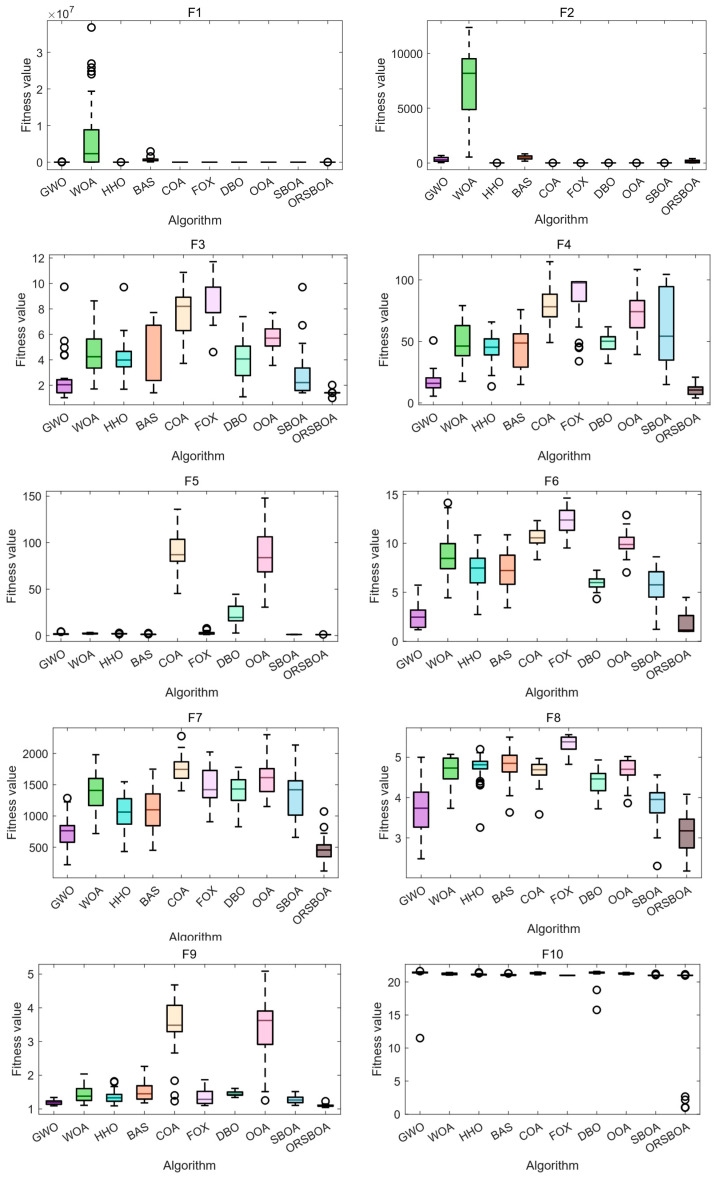
Boxplot comparison of ORSBOA with other algorithms on CEC2019.

Figure 5 shows the boxplots of the algorithms on functions F1~F10, further illustrating the concentration and stability of the solution distribution. It is observed that ORSBOA has a significantly smaller range of box lines than most of the compared algorithms, which indicates lower variability and greater stability of the solution. Shorter whiskers and fewer outliers reflect the centralized distribution and controlled fluctuations of the solution.

Especially in functions such as F9 and F10, WOA algorithm and COA, etc. show obvious outliers and divergent solutions, while ORSBOA always maintains good stability and consistency, which highlights the robustness of ORSBOA in dealing with complex nonlinear optimization problems.

In summary, based on the multi-dimensional experimental results and statistical analysis, ORSBOA demonstrates excellent global optimization seeking ability, fast and stable convergence behavior, and significant robustness on CEC2019 benchmark functions, which significantly outperforms a variety of mainstream population intelligence optimization algorithms, including SBOA. These results validate the effectiveness of the proposed algorithmic improvement and lay a solid foundation for the practical application and further extension of ORSBOA in engineering optimization problems.

### 3.3. CEC2022 Experimental Results

In order to further verify the generality and robustness of ORSBOA in solving highly complex optimization problems, the CEC2022 benchmark function set is selected as the experimental platform. The function set contains 12 representative objective functions covering multi-peak, composite and hybrid types. These functions are complex in design and diverse in nature, and are widely used for evaluating algorithmic performance for high-dimensional, multi-peaked and irregular search space problems.

**Figure 6:**
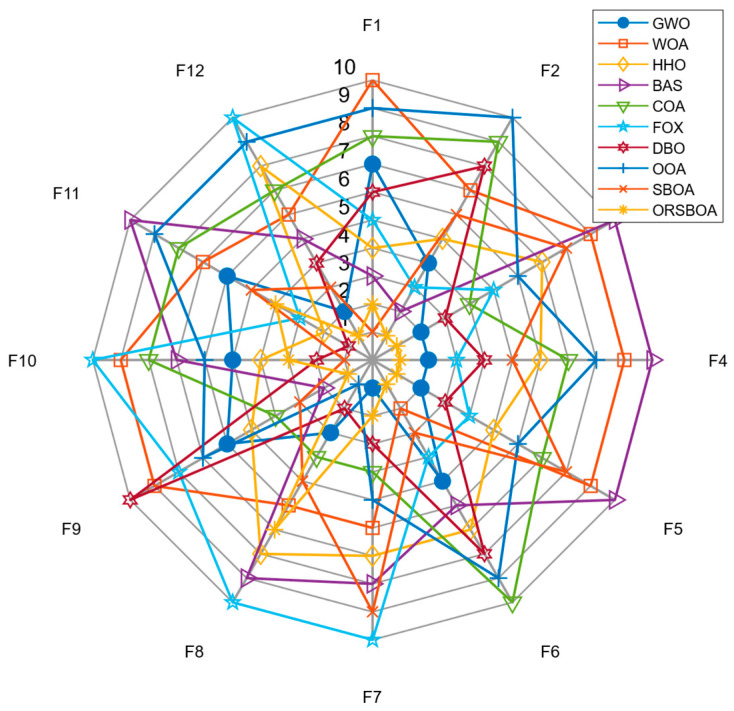
Radar diagram of ORSBOA and comparison algorithm on CEC2022.

**Figure 7:**
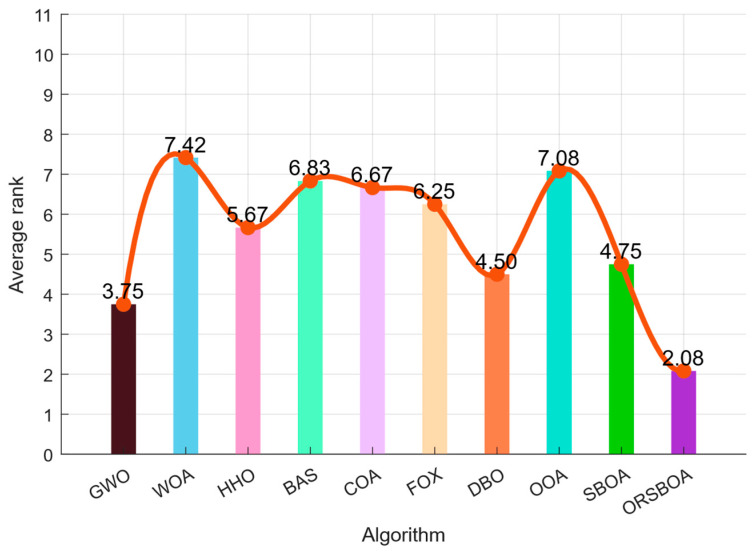
Plot of average ranking of ORSBOA and comparison algorithms on CEC2022.

In terms of overall ranking performance, ORSBOA leads on most functions. The radar chart in Figure 6 shows the average ranking of each algorithm on functions F1~F12, and the ranking of ORSBOA is always located near the center, which indicates that it dominates the fitness optimization. The average ranking bar chart in Figure 7 shows that ORSBOA has an average ranking of 2.08, which is significantly better than several mainstream algorithms such as DBO (3.75), FOX (4.50), and HHO (5.08), as well as significantly better than the algorithms such as WOA (7.25) and BAS (7.08), etc. The average ranking of ORSBOA is 4.75 for this function set, which is moderate, but still lower than the improved ORSBOA, which has an average ranking of 4.75, and has a better performance than the improved ORSBOA. moderate, but still lower than the improved ORSBOA, thus verifying the effectiveness of the proposed hybrid strategy in improving the performance of the algorithm.

From the numerical statistics in Table 4, it can be seen that ORSBOA achieves the lowest mean and standard deviation on a number of functions such as F1, F2, F6, and F10, demonstrating excellent search accuracy and stability. For example, on the F6 function, ORSBOA significantly outperforms HHO, DBO, and GWO, not only obtaining a lower average fitness value, but also obtaining the smallest solution variance. Meanwhile, its best fitness value remains excellent on several functions, further highlighting its accuracy and consistency in global optimal search.

In terms of statistical significance, the results in Table 5 and Table 6 show that ORSBOA exhibits a significant difference in optimization performance from the other algorithms on the vast majority of the tested functions. *p*-values are generally lower than 0.05, and the significance level of some of the results is even as low as $10^{-6}$. Notably, in the pairwise comparison with SBOA, ORSBOA achieves statistical superiority on 10 out of 12 functions, further confirming the effectiveness of combining the optimal neighborhood perturbation and the Reverse learning strategy in enhancing the optimization performance.

**Figure 8:**
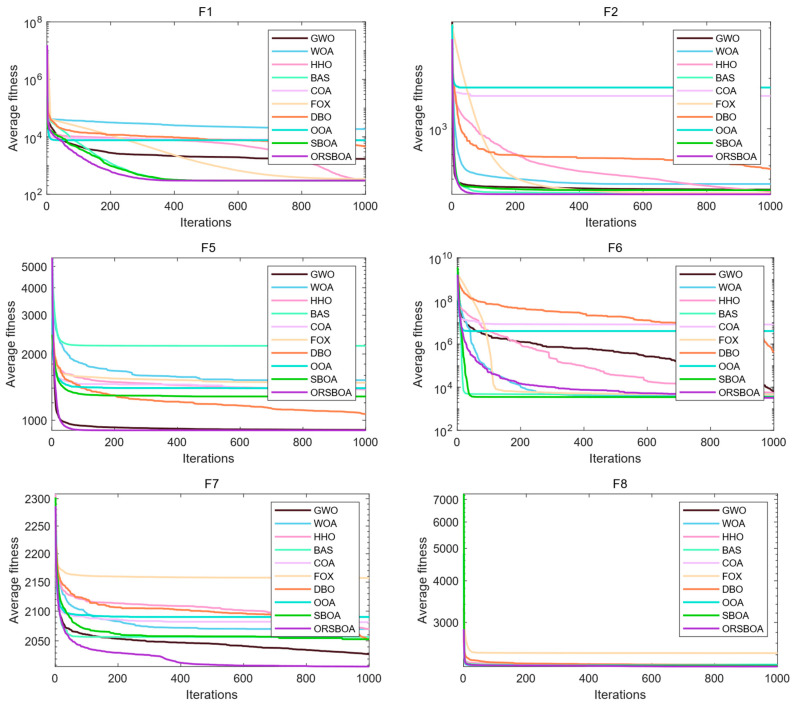

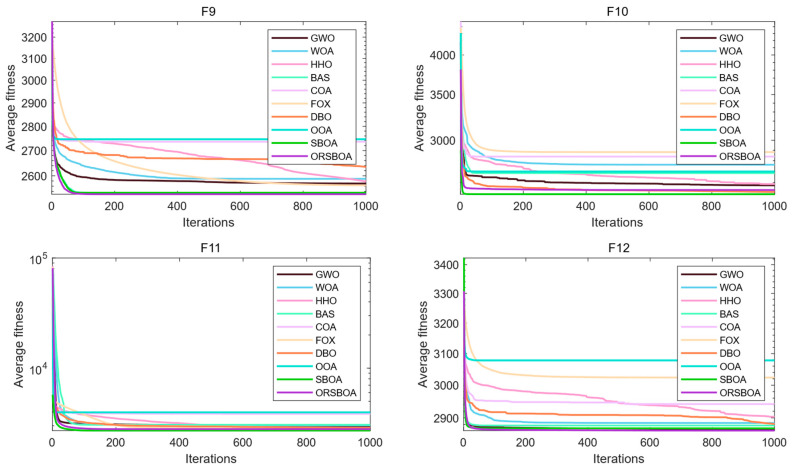
Average convergence plot of ORSBOA and comparison algorithms on CEC2022.

**Figure 9:**
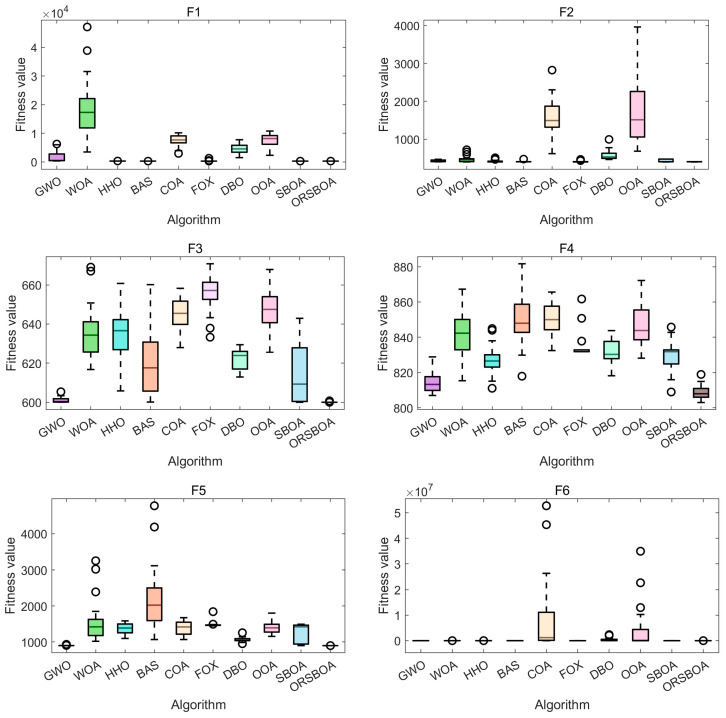

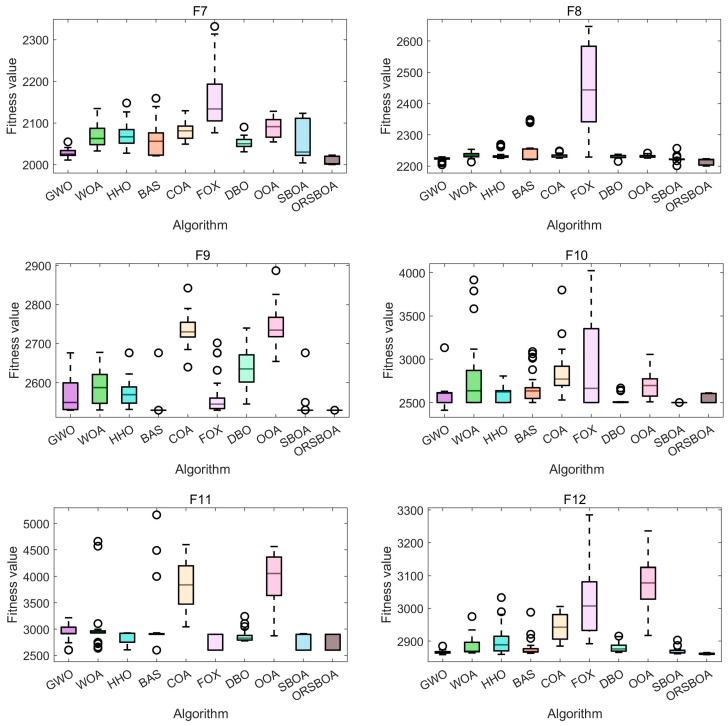
Boxplot comparison of ORSBOA with other algorithms on CEC2022.

Figure 8 shows the average convergence curves of the algorithm on the CEC2022 benchmark functions, providing a visual assessment of the algorithm’s speed of convergence and utilization capabilities. In terms of trends, ORSBOA exhibits a fast fitness degradation on most of the functions. Notably, for complex functions such as F1 and F6, the fitness value decreases significantly in the first 200 iterations and then maintains a steady decrease before finally converging to the neighborhood of the global optimum. In contrast, algorithms such as WOA and FOX have large fluctuations during the convergence process, and the search behavior is not so stable, resulting in the overall convergence performance not as good as that of ORSBOA. The box plots in Figure 9 further illustrate the robustness of the algorithms in terms of solution distribution. It can be seen that ORSBOA exhibits a compact box-and-line distribution and shorter whiskers for most functions, which indicates lower variability, fewer outliers and more concentrated solutions. In contrast, algorithms such as COA and WOA exhibit significant skewness and outliers on several functions, indicating that they are less stable and the optimization results are more sensitive to fluctuations in the initial population setting and search strategy.

Through multi-perspective performance analysis and statistical validation, ORSBOA shows excellent global search capability, good convergence stability and significant robustness on the CEC2022 test function set, and its overall performance exceeds that of SBOA and other mainstream optimization algorithms, which further validates its wide adaptability and practical value in solving high-dimensional complex optimization problems.

Although a separate ablation study was not performed, careful inspection of the convergence behaviors and performance trends reveals the complementary roles of ONP and RLS. ONP primarily enhances global exploration and robustness, as reflected in the improved performance on multimodal functions in CEC2019, while RLS mainly improves convergence accuracy and exploitation ability, as seen in the hybrid and composition functions in CEC2022. These observations confirm that the two strategies contribute differently yet synergistically to the overall effectiveness of ORSBOA.

### 3.4. Application of ORSBOA in Engineering Optimization Problems

#### 3.4.1. Welded Beam

The objective of the welded beam structural optimization problem is to minimize the manufacturing cost while satisfying the engineering constraints such as strength and stiffness. The experimental results are shown in Table 7. ORSBOA achieves significant performance advantages in all the metrics. Its optimal solution is 1.670218, and the worst solution is only 1.670254, with a very small fluctuation range, which reflects the excellent stability and anti-interference ability of the algorithm. In addition, the average and median solutions are 1.670225 and 1.670221, respectively, which are almost the same as the optimal solution, indicating that the algorithm shows good convergence consistency in multiple independent runs.

In terms of robustness, the standard deviation of ORSBOA is only $1.17\times 10^{-5}$, which is significantly lower than that of HHO (0.224695), BAS (0.128668) and OOA (0.415338), which further verifies its stable performance in engineering optimization.

In terms of the average computation time, ORSBOA has a computation time of 0.227155 s, which is slightly higher than that of GWO (0.115776) and FOX (0.139073), but still lower than that of SBOA (0.392841) and OOA (0.219622). Considering the solution quality and computational efficiency, ORSBOA ensures the accuracy and stability while maintaining a reasonable computational cost, showing a strong value for engineering applications. In terms of statistical significance tests, the Wilcoxon signed-rank test shows that ORSBOA is significantly different from all other algorithms, and its *p*-value is always well below the 0.05 significance threshold. For example, ORSBOA reaches extremely significant levels compared to WOA ($2.69\times 10^{-75}$), HHO ($4.1\times 10^{-99}$), COA ($2.7\times 10^{-96}$) and DBO ($1.18\times 10^{-98}$). These results clearly show that the superior performance of ORSBOA on this problem is not only numerically superior but also statistically robust.

The Wilcoxon rank sum test also shows that all *p*-values are lower than $10^{-120}$ when comparing ORSBOA to HHO, BAS, FOX, and DBO, suggesting that the performance improvements realized by ORSBOA are systematic and not due to randomness.

**Figure 10:**
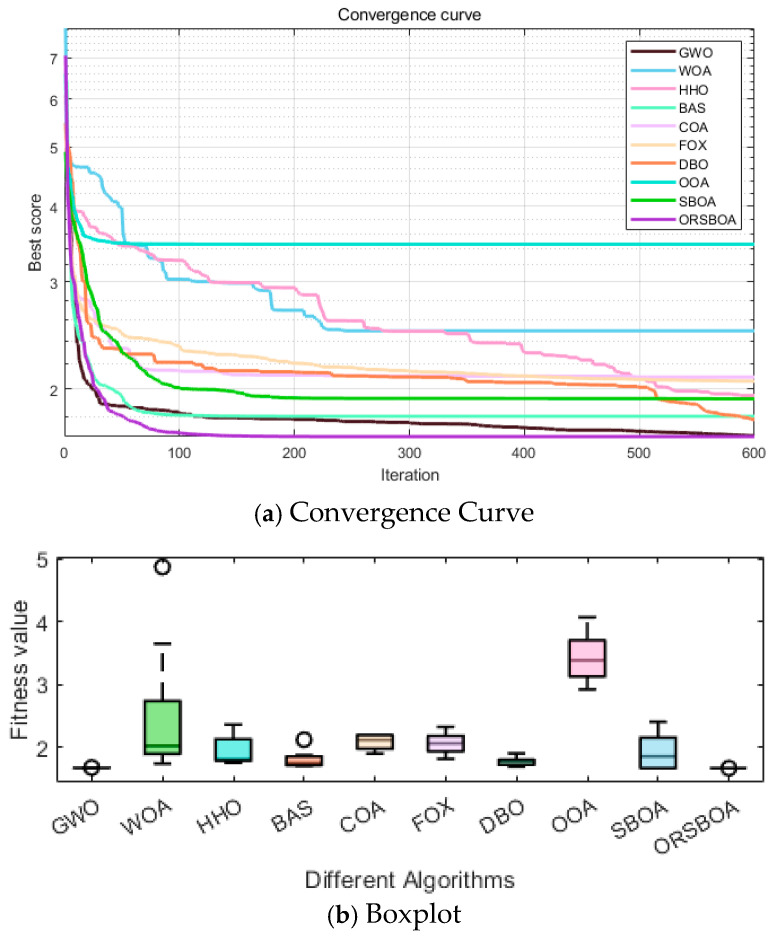
Convergence curves and boxplots of ORSBOA vs. comparison algorithms for the welded beam design problem.

The convergence efficiency of ORSBOA is further verified by the average convergence curve shown in Figure 10a. From the figure, it can be seen that ORSBOA shows a rapid decreasing trend at the beginning of the iteration, which is significantly faster than the comparison algorithms such as WOA, FOX and COA. ORSBOA stabilizes rapidly near the global optimum within a relatively small number of iterations, which indicates that it has a strong global searching ability and a high local utilization accuracy.

Figure 10b shows the boxplots of the algorithms to demonstrate the stability of the solutions from the distributional perspective. The boxplots of ORSBOA are extremely compact in range, with minimal fluctuations and no obvious outliers, indicating that the distribution of the solutions is highly concentrated and exhibits excellent robustness and consistency.

#### 3.4.2. Three-Bar Truss

The objective of the three-bar truss design problem is to minimize the weight of the structure while satisfying the stress, geometry and material strength constraints. This is a classical multivariate constrained structural optimization problem. As shown in Table 8, ORSBOA consistently exhibits excellent performance on this problem. Its optimal solution of 263.895984 outperforms the best results of all the compared algorithms, indicating its significant advantage in global optimization. In addition, its worst solution is 263.8963 and its average solution is 263.8959, with a very small difference between the two, indicating that ORSBOA obtains consistent and stable results over multiple independent runs.

Its standard deviation is only $1.52\times 10^{-4}$, which is much lower than that of WOA (4.2413) and OOA (2.155156), which further confirms that ORSBOA’s results have minimal fluctuation and stable convergence. In terms of computational efficiency, the average execution time of ORSBOA is 0.204208 s, which is better than that of HHO, BAS, COA, OOA, and SBOA, indicating that it maintains a high solution quality while ensuring strong runtime performance. In terms of statistical significance, the Wilcoxon signed-rank test results show that the *p*-value of ORSBOA compared to the other algorithms is well below the 0.05 significance threshold. In addition, the rank sum test results show that the *p*-value of ORSBOA is $5.8\times 10^{-156}$, which is significantly lower than the mean of the compared algorithms, verifying the high statistical reliability of the performance advantage of ORSBOA on this problem.

**Figure 11:**
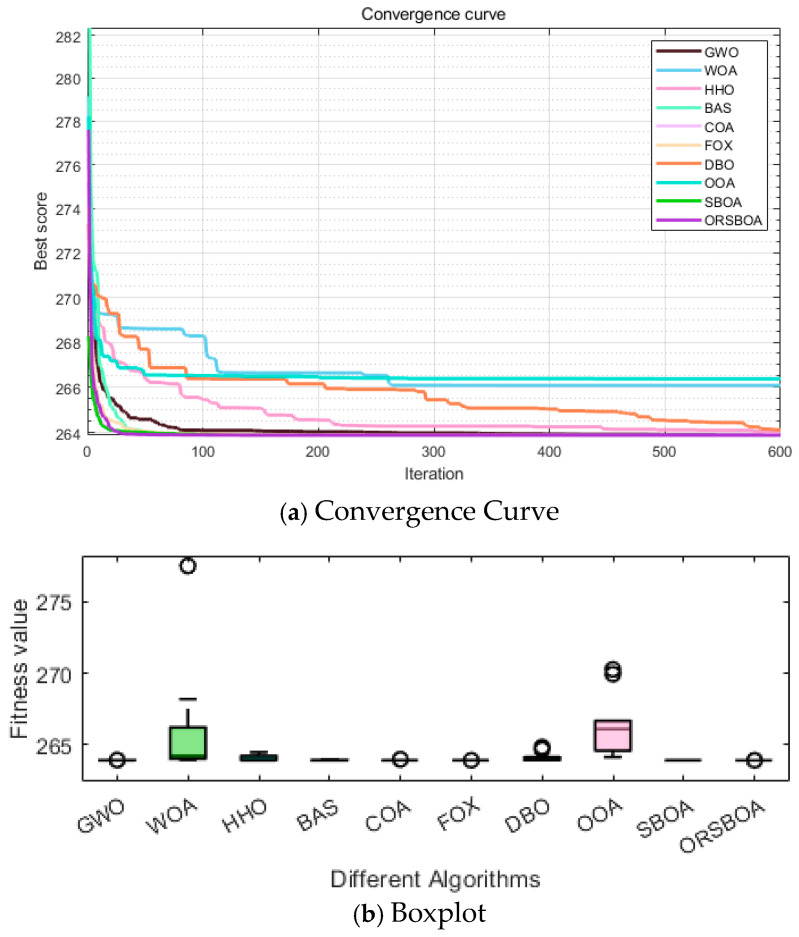
Convergence Curve and Boxplot Comparisons of ORSBOA and Comparative Algorithms on the Three-Bar Truss Design Problem.

The average convergence curve shown in Figure 11a clearly indicates that ORSBOA rapidly reduces the objective function value in the early stage of optimization and maintains a stable downward trend in the later stage, eventually converging to a high-quality solution. In contrast, some comparison algorithms exhibit oscillatory behavior or premature convergence, resulting in less stable performance.

The corresponding boxplots are given in Figure 11b, further verifying the robustness advantage of ORSBOA. As can be seen from the figure, ORSBOA has the narrowest box line range and the most concentrated distribution of results with almost no outliers. Its median is also very close to the optimal solution, indicating that ORSBOA shows strong consistency over multiple runs, demonstrating the algorithm’s high robustness and practicality.

#### 3.4.3. Cantilever Beam

The objective of the cantilever beam design problem is to minimize the weight of the structure while satisfying the physical constraints such as stress and deflection, which is a typical engineering optimization problem. As shown in Table 9, ORSBOA exhibits significant performance advantages in this problem. ORSBOA outperforms all other compared algorithms in all three key accuracy metrics—minimum, mean and median. Specifically, it achieves the lowest minimum value of 1.339957 among all methods, and the optimal solution is close to that value; its standard deviation is only $3.51\times 10^{-6}$, which is much lower than that of WOA, HHO, and BAS, indicating that the solution distribution is extremely concentrated over multiple independent runs, demonstrating excellent optimization stability. The worst solution is 1.339968, which also remains at a high quality level, further proving the excellent robustness and reliability of ORSBOA.

In terms of computational efficiency, although ORSBOA integrates multiple strategy mechanisms, its average running time is 0.069479 s, which is slightly higher than some lightweight algorithms but still lower than methods such as BAS (0.122796 s). This suggests that ORSBOA effectively balances the quality of the solution and computational efficiency, striking a good balance between accuracy and time cost.

In terms of statistical significance, the results of both Wilcoxon signed rank test and rank sum test confirm the statistical superiority of the optimization results of ORSBOA. Compared with algorithms such as GWO, WOA, HHO and COA, the *p*-value of the ORSBOA is consistently well below the significance threshold of 0.05, with a minimum value of $1.58\times 10^{-61}$. This indicates the significant and statistically reliable performance advantage of ORSBOA in solving cantilever beam design problems.

**Figure 12:**
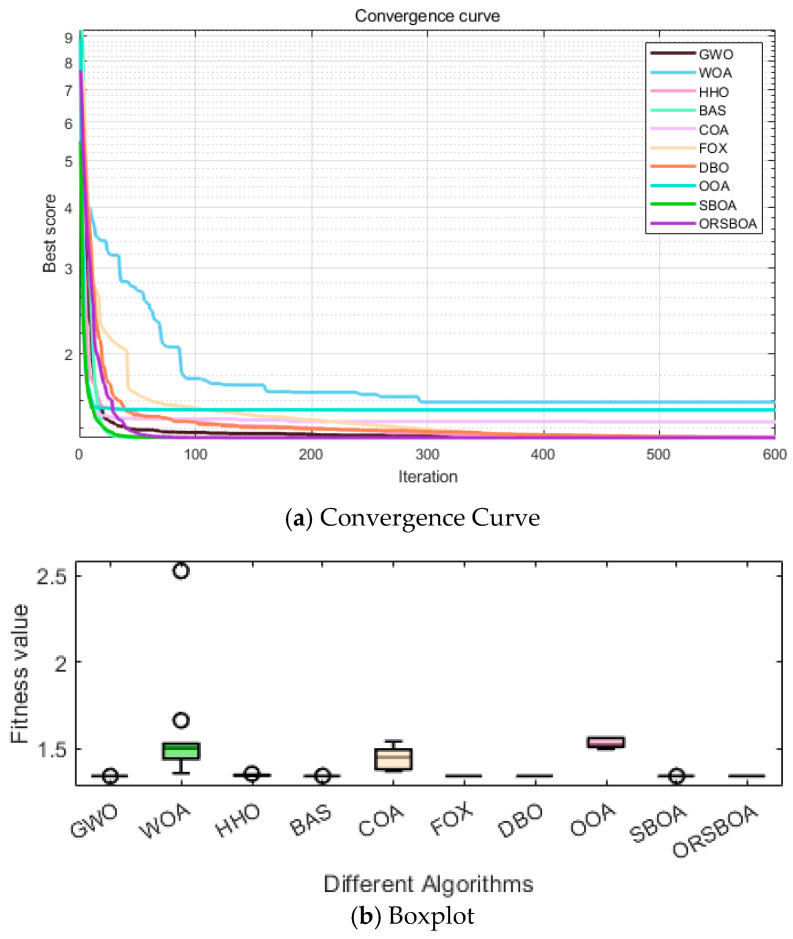
Convergence curves and boxplots of ORSBOA vs. comparative algorithms for the cantilever beam design problem.

As shown in the average convergence curve in Figure 12a, ORSBOA converges rapidly at the beginning of the iteration, and is close to the optimal solution within the first 100 iterations. The subsequent optimization process remains stable with small fluctuations, showing strong global exploration and local exploitation capabilities.

The box plot in Figure 12b further supports the stability advantage of ORSBOA from the perspective of the distribution of the results, and the narrower range and fewer outliers of ORSBOA indicate that its solution concentration and consistency are better than that of the other algorithms. This advantage is especially evident when compared to WOA, COA and OOA.

#### 3.4.4. Compression Spring

The objective of the compression spring design problem is to minimize the total manufacturing cost while strictly satisfying multiple constraints such as material strength, geometry and manufacturing feasibility. As shown in the statistical results in Table 10, ORSBOA achieves excellent performance on this problem. Its Min value reaches 0.012666, which is optimal among all algorithms, comparable to methods such as BAS and FOX, and superior to conventional algorithms such as WOA (0.012813) and FOX (0.012677).

In terms of Worse value, ORSBOA reaches 0.012729, which is not absolutely optimal but significantly better than algorithms with convergence instability problems such as COA (0.021509) and OOA ($6.32\times 10^{14}$), thus proving the superior optimization stability of ORSBOA.

In terms of solution concentration and variability, the standard deviation and median of ORSBOA are $2.09\times 10^{-5}$ and 0.012691, respectively, indicating that the solutions are highly concentrated in multiple runs and the results are tightly clustered around the optimal value, which reflects strong robustness. The average value of 0.012695 is also lower than most of the compared algorithms, indicating that ORSBOA not only performs well in a single run, but also maintains a highly stable optimization performance in the whole.

In terms of computational efficiency, the average running time of ORSBOA is 0.229789 s, which is slightly higher than that of lightweight algorithms such as GWO (0.118662) and WOA (0.14385), but is still within the acceptable range, especially considering its accuracy and convergence stability, which makes it valuable for practical application in engineering applications.

In terms of statistical significance, both the Wilcoxon signed rank test and the rank sum test results show that ORSBOA has a significant statistical advantage over most algorithms. The *p*-values of the signed rank test for comparisons with WOA, HHO, COA, and OOA were all well below the threshold of 0.05, with the lowest being reached $6.7\times 10^{-102}$. This is further confirmed by the results of the rank sum test, e.g., the *p*-value of $1.02\times 10^{-173}$ for comparison with DBO, which clearly demonstrates that ORSBOA is able to maintain a stable and reliable optimization performance under complex constraint environments.

**Figure 13:**
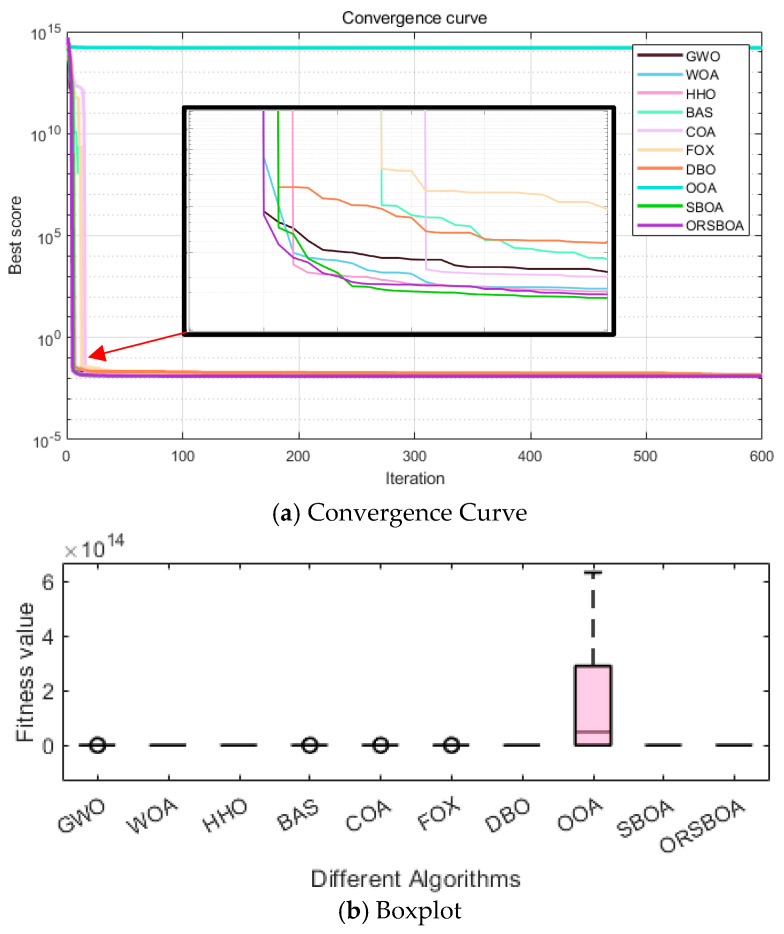
Convergence curves and box plots of ORSBOA compared to the comparison algorithm for the compression spring design problem.

The average convergence curve in Figure 13a clearly shows that ORSBOA declines rapidly at the beginning of the iteration and approaches the global optimum region quickly. In the later stages, ORSBOA maintains a stable convergence, outperforming algorithms such as WOA and COA that exhibit significant oscillations and premature convergence trends.

The robustness of the algorithm is further confirmed by the boxplot in Figure 13b, where ORSBOA shows a compact distribution with few outliers and a median value close to the optimum, indicating that the algorithm consistently delivers stable results over multiple experiments.

## 4. Discussion

ORSBOA enhances SBOA in global exploration, local exploitation, and convergence stability through the coordinated action of ONP and RLS. Extensive experiments on CEC2019/CEC2022 and classical engineering cases consistently show gains in solution quality, robustness, and efficiency.

Mechanistic insight: ONP applies small, directed perturbations within a shrinking neighborhood of elite solutions, intensifying local refinement while preserving guidance and diversity. RLS generates opposite candidates that expand coverage and counteract early stagnation. Their complementary roles produce a stable exploration–exploitation balance, which is especially beneficial on multimodal, high-dimensional landscapes.

Benchmark evidence: On both suites, ORSBOA attains competitive or superior Min/Avg/Median with lower Std on most functions, indicating accuracy and run-to-run stability. Wilcoxon rank-sum and signed-rank tests corroborate these improvements, supporting their statistical reliability.

Engineering relevance: On welded beam, three-bar truss, cantilever beam, and compression spring problems—characterized by nonlinear objectives, tight constraints, and strong variable coupling—ORSBOA yields higher-quality designs, faster convergence, and tighter solution concentration than the baselines, demonstrating practicality for structural optimization.

Parameter sensitivity and trade-offs: Performance is influenced by the decay rate of the ONP neighborhood radius and the perturbation probability. Slower decay prolongs diversity and improves escape ability but can slow convergence; faster decay accelerates convergence yet increases stagnation risk. Higher perturbation probabilities enhance robustness across runs, whereas lower values favor efficiency at the expense of stability. A systematic sensitivity analysis will be pursued to quantify these effects.

Computational considerations: Due to ONP/RLS operations, ORSBOA incurs moderately higher overhead than lightweight methods such as GWO and BAS, though the overall complexity remains O(N⋅D⋅T). When runtime is paramount, lightweight baselines may be preferable; otherwise, ORSBOA’s accuracy and robustness justify the additional cost.

Relation to recent hybrids: Unlike OBL–local-search hybrids that employ static triggers or independent operators, ORSBOA co-designs ONP and RLS with adaptive scheduling, yielding emergent benefits beyond simple aggregation and reducing susceptibility to premature convergence.

Limitations and outlook: In very high dimensions, diversity can erode, raising the risk of premature convergence. The current parameterization is static, which may limit adaptability in dynamic environments. Extensions to multi-objective, dynamic, and combinatorial settings remain to be verified. Future work will focus on (i) adaptive/self-tuning control of ONP/RLS, (ii) efficient variants for large-scale problems (e.g., sparse neighborhoods, surrogate assistance, selective operator invocation), and (iii) validation on more complex engineering tasks (e.g., multidisciplinary or heavily constrained designs).

Conclusion of discussion: ORSBOA offers a principled, empirically validated dual-mechanism enhancement for swarm optimization, improving robustness and solution quality on challenging benchmarks and engineering tasks while maintaining practical scalability.

## 5. Conclusions

A dual-mechanism enhanced secretary bird optimization algorithm, ORSBOA, was presented by integrating ONP and RLS to balance global exploration and local exploitation. Across CEC2019/CEC2022 and four classical engineering design problems, ORSBOA outperformed nine representative metaheuristics in convergence accuracy, robustness, and stability; nonparametric tests confirmed the statistical significance of these gains. The hybrid ONP–RLS framework provides actionable guidance for designing robust swarm optimizers and demonstrates practical value for complex engineering optimization.

Notwithstanding these strengths, ORSBOA entails moderately higher computational cost than lightweight baselines and may lose diversity in very high dimensions under static settings. Future research will explore adaptive parameter control, efficient implementations for large-scale scenarios, and extensions to dynamic and multi-objective optimization, as well as applications to real-time engineering tasks. Beyond performance improvements, this work highlights how complementary, biomimetic strategies—neighborhood interaction and reverse learning—can be co-designed to yield resilient population-based search.

## Figures and Tables

**Table 1:** Statistical summary of six performance metrics for ORSBOA vs. comparator algorithms on the CEC2019.

Function	Algorithm	Min	Std	Avg	Median	Worse	Avg_Time
F1	GWO	1	26,433.2753	8288.96925	97.57998955	141,253.6354	0.087453443
	WOA	129.2964496	10,529,798.99	7,477,008.716	2,327,922.044	36,819,291.15	0.11251931
	HHO	1	4.49137 × 10^−7^	1.000000082	1	1.00000246	0.16478975
	BAS	59,971.75775	526,347.8788	705,467.7571	683,155.6799	2,932,674.326	0.292612313
	COA	1	0	1	1	1	0.170127907
	FOX	1	0	1	1	1	8.361275527
	DBO	1	0	1	1	1	0.126512527
	OOA	1	0	1	1	1	0.141128803
	SBOA	1	0	1	1	1	0.406519323
	ORSBOA	1	6989.568306	2235.106844	23.11011601	33,696.17608	0.15925187
F2	GWO	29.98405	197.6724	323.7175	293.8009	675.4894	0.077898
	WOA	536.5078	3017.083	7232.377	8181.026	12,365.04	0.070319
	HHO	4.65392	0.063185	4.988464	5	5	0.104457
	BAS	161.9812	171.6015	506.9483	496.664	828.3378	0.173416
	COA	4.99185	0.001488	4.999728	5	5	0.068273
	FOX	4.289288	0.198548	4.932887	5	5	0.05904
	DBO	4.219359	0.294602	4.403294	4.273456	5	0.080479
	OOA	4.999986	2.63 × 10^−6^	5	5	5	0.065451
	SBOA	4.216916	0.232084	4.923958	5	5	0.283605
	ORSBOA	4.272532	118.4001	141.7239	136.6592	396.7099	0.081015
F3	GWO	1.023801	1.799592	2.444966	2.04261	9.735138	0.078849
	WOA	1.709302	1.784579	4.565346	4.245001	8.630907	0.067947
	HHO	1.689426	1.485829	4.186346	3.98517	9.710329	0.104101
	BAS	1.409135	2.464254	5.314404	6.710876	7.71202	0.162917
	COA	3.720018	1.608286	7.756434	8.20077	10.87069	0.067685
	FOX	4.608729	1.60571	8.984601	9.707786	11.71071	0.059407
	DBO	1.094176	1.426182	3.937853	4.069862	7.39227	0.085547
	OOA	3.557997	1.015331	5.741795	5.702818	7.717422	0.068586
	SBOA	1.409136	1.864101	2.874209	2.211908	9.709991	0.308667
	ORSBOA	1	0.136481	1.415887	1.409135	2.020819	0.08511
F4	GWO	5.446891	8.63154	17.24045	15.93122	50.74477	0.068501
	WOA	17.53902	15.07645	48.46255	46.21035	79.10805	0.078456
	HHO	13.40423	11.76786	44.34437	45.22134	65.80176	0.124643
	BAS	14.92941	19.07911	43.9771	48.69016	75.82988	0.192088
	COA	49.09412	16.01102	79.84698	78.17986	114.8192	0.094186
	FOX	33.83358	19.69164	86.03069	97.50973	98.5047	0.069634
	DBO	32.08445	7.909186	49.10821	50.12904	61.83827	0.094337
	OOA	39.35627	16.62678	73.39555	74.17606	108.4939	0.089693
	SBOA	14.92942	28.88922	62.13807	54.22997	104.4744	0.331282
	ORSBOA	3.984877	4.424557	10.45218	10.45211	20.89915	0.098373
F5	GWO	1.192799	0.826558	1.788975	1.634912	4.338824	0.072657
	WOA	1.419477	0.483545	2.186047	2.101362	3.286566	0.09114
	HHO	1.511363	0.262718	1.99488	1.979652	2.834647	0.151598
	BAS	1.061448	0.302046	1.270137	1.207782	2.673017	0.233409
	COA	45.35443	21.69732	87.65408	86.94503	135.9153	0.113493
	FOX	1.069055	1.814755	2.773019	2.197543	7.785705	0.087926
	DBO	2.736627	9.64848	22.90427	19.52394	44.49723	0.116008
	OOA	30.5915	30.94	85.58133	83.75194	148.0068	0.112845
	SBOA	1.029552	0.05023	1.135997	1.135359	1.229125	0.38968
	ORSBOA	1	0.03663	1.03657	1.029562	1.187215	0.113431
F6	GWO	1.171681	1.224139	2.598437	2.458291	5.737872	0.71326
	WOA	4.440269	2.123321	8.90316	8.451798	14.14377	0.749521
	HHO	2.729712	1.978913	7.185723	7.463491	10.8432	1.71824
	BAS	3.421003	2.043268	7.209984	7.207905	10.86966	2.175535
	COA	8.322128	0.979707	10.54156	10.55832	12.32501	1.741269
	FOX	9.518888	1.364651	12.25772	12.38127	14.63156	0.730659
	DBO	4.318829	0.671776	5.959194	6.00107	7.238252	0.758716
	OOA	7.012894	1.169093	10.02777	9.879509	12.89007	1.402222
	SBOA	1.209526	1.99107	5.647841	5.769201	8.612215	1.465639
	ORSBOA	1.000016	1.014493	1.7938	1.133314	4.478898	1.395455
F7	GWO	220.0029	251.7331	743.1388	763.3723	1285.952	0.073353
	WOA	719.0708	331.4385	1403.811	1408.39	1980.332	0.086493
	HHO	431.4434	296.3624	1085.482	1062.625	1547.103	0.1408
	BAS	451.2545	330.4752	1109.455	1099.037	1749.247	0.259545
	COA	1402.694	204.0641	1747.11	1745.585	2277.196	0.11486
	FOX	907.5505	289.9357	1487.829	1420.761	2024.104	0.075744
	DBO	827.6562	234.4982	1408.781	1431.365	1777.967	0.111061
	OOA	1151.132	293.792	1641.282	1613.593	2300.111	0.100606
	SBOA	657.9357	375.9359	1317.354	1419.456	2134.733	0.331941
	ORSBOA	120.0184	202.6388	471.1316	455.4021	1071.329	0.106162
F8	GWO	2.48143	0.615267	3.721255	3.736851	5.000801	0.068071
	WOA	3.733717	0.372582	4.648361	4.733284	5.073549	0.080508
	HHO	3.256688	0.357256	4.736858	4.815837	5.200854	0.129863
	BAS	3.632055	0.38745	4.820542	4.849939	5.500841	0.196355
	COA	3.581649	0.288453	4.640778	4.69199	4.971196	0.093595
	FOX	4.825455	0.200885	5.334997	5.383339	5.561268	0.069
	DBO	3.721357	0.306351	4.378851	4.462824	4.93377	0.103154
	OOA	3.863427	0.289764	4.691326	4.703193	5.019631	0.09142
	SBOA	2.303621	0.506524	3.882274	3.953864	4.561328	0.322409
	ORSBOA	2.178231	0.460091	3.13597	3.174504	4.08238	0.094795
F9	GWO	1.092502	0.068378	1.192074	1.189192	1.340142	0.061125
	WOA	1.107368	0.250364	1.447341	1.378932	2.036444	0.073696
	HHO	1.091539	0.17791	1.364388	1.330881	1.817725	0.104165
	BAS	1.179269	0.291526	1.513998	1.44924	2.262245	0.164895
	COA	1.230104	0.848112	3.486511	3.483143	4.678878	0.073844
	FOX	1.100777	0.223092	1.345754	1.284683	1.866036	0.059623
	DBO	1.339881	0.072649	1.46083	1.439658	1.60841	0.083912
	OOA	1.252036	0.875559	3.439671	3.623511	5.0865	0.074239
	SBOA	1.10489	0.103351	1.27429	1.264632	1.515009	0.308023
	ORSBOA	1.043066	0.033285	1.100551	1.094945	1.227533	0.084388
F10	GWO	11.50068	1.814723	21.10049	21.4367	21.6318	0.070258
	WOA	21.03613	0.125617	21.21531	21.21428	21.43693	0.083063
	HHO	20.98578	0.094978	21.13226	21.12721	21.44901	0.133685
	BAS	20.99615	0.094914	21.05718	21	21.30317	0.194076
	COA	21.07793	0.11283	21.30825	21.31495	21.51088	0.098854
	FOX	20.98244	0.00742	20.98983	20.98564	21.00011	0.069132
	DBO	15.76889	1.131014	21.16186	21.46239	21.5926	0.097964
	OOA	21.07947	0.096514	21.26044	21.25594	21.44108	0.093477
	SBOA	20.97191	0.05923	21.00692	20.99007	21.24199	0.316781
	ORSBOA	1	7.957874	17.10338	21.00032	21.15426	0.096224

**Table 2:** Wilcoxon signed-rank test results for ORSBOA vs. comparator algorithms on the CEC2019.

Function	WOA	HHO	BAS	COA	FOX	DBO	OOA	SBOA	ORSBOA
F1	3.51524 × 10^−6^	1.7344 × 10^−6^	1.7344 × 10^−6^	1.7344 × 10^−6^	1.7344 × 10^−6^	1.7344 × 10^−6^	1.7344 × 10^−6^	1.7344 × 10^−6^	0.003161765
F2	1.7344 × 10^−6^	1.7344 × 10^−6^	8.91873 × 10^−5^	1.7344 × 10^−6^	1.7344 × 10^−6^	1.7344 × 10^−6^	1.7344 × 10^−6^	1.7344 × 10^−6^	0.000453356
F3	0.000114992	0.00024118	0.000388111	1.92092 × 10^−6^	1.92092 × 10^−6^	0.002414704	1.97295 × 10^−5^	0.338856155	0.000189097
F4	2.35342 × 10^−6^	2.35342 × 10^−6^	1.36011 × 10^−5^	1.7344 × 10^−6^	1.7344 × 10^−6^	1.92092 × 10^−6^	1.92092 × 10^−6^	4.7292 × 10^−6^	4.44934 × 10^−5^
F5	0.006835856	0.005667173	0.000135948	1.7344 × 10^−6^	0.004114031	1.7344 × 10^−6^	1.7344 × 10^−6^	1.92092 × 10^−6^	1.7344 × 10^−6^
F6	1.7344 × 10^−6^	2.8786 × 10^−6^	1.7344 × 10^−6^	1.7344 × 10^−6^	1.7344 × 10^−6^	2.12664 × 10^−6^	1.7344 × 10^−6^	4.7292 × 10^−6^	0.009842142
F7	5.75165 × 10^−6^	0.000663921	0.000570644	1.7344 × 10^−6^	1.7344 × 10^−6^	1.92092 × 10^−6^	1.7344 × 10^−6^	3.18168 × 10^−6^	0.000174228
F8	3.88218 × 10^−6^	2.35342 × 10^−6^	2.12664 × 10^−6^	6.33914 × 10^−6^	1.7344 × 10^−6^	0.000105695	2.8786 × 10^−6^	0.245190309	7.51366 × 10^−5^
F9	2.59671 × 10^−5^	3.11232 × 10^−5^	1.79885 × 10^−5^	1.7344 × 10^−6^	0.006835856	1.7344 × 10^−6^	1.92092 × 10^−6^	0.00089443	2.60333 × 10^−6^
F10	3.11232 × 10^−5^	4.07151 × 10^−5^	3.11232 × 10^−5^	0.00089443	3.11232 × 10^−5^	0.829013032	0.000125057	3.11232 × 10^−5^	1.79885 × 10^−5^

**Table 3:** Wilcoxon rank-sum test results for ORSBOA vs. comparator algorithms on the CEC2019.

Function	WOA	HHO	BAS	COA	FOX	DBO	OOA	SBOA	ORSBOA
F1	4.99795 × 10^−9^	3.01586 × 10^−12^	4.07716 × 10^−11^	1.21178 × 10^−12^	1.21178 × 10^−12^	1.21178 × 10^−12^	1.21178 × 10^−12^	1.21178 × 10^−12^	0.024156885
F2	4.97517 × 10^−11^	1.72025 × 10^−12^	0.001173758	2.36567 × 10^−12^	4.11097 × 10^−12^	3.01797 × 10^−11^	3.15782 × 10^−12^	3.16021 × 10^−12^	0.000283887
F3	5.46203 × 10^−6^	4.7445 × 10^−6^	0.001057554	7.38029 × 10^−10^	3.82016 × 10^−10^	1.52917 × 10^−5^	4.1825 × 10^−9^	0.162375022	2.19589 × 10^−7^
F4	7.38029 × 10^−10^	4.1825 × 10^−9^	6.01039 × 10^−8^	3.33839 × 10^−11^	4.50432 × 10^−11^	1.77691 × 10^−10^	4.50432 × 10^−11^	1.06735 × 10^−9^	0.000104066
F5	3.15727 × 10^−5^	7.22083 × 10^−6^	3.09389 × 10^−6^	3.01986 × 10^−11^	0.012732115	4.07716 × 10^−11^	3.01986 × 10^−11^	6.06576 × 10^−11^	3.01986 × 10^−11^
F6	4.07716 × 10^−11^	6.72195 × 10^−10^	2.60985 × 10^−10^	3.01986 × 10^−11^	3.01986 × 10^−11^	1.46431 × 10^−10^	3.01986 × 10^−11^	1.59641 × 10^−7^	0.001952677
F7	5.46175 × 10^−9^	2.59736 × 10^−5^	2.59736 × 10^−5^	3.01986 × 10^−11^	3.82016 × 10^−10^	5.07231 × 10^−10^	5.49405 × 10^−11^	1.35943 × 10^−7^	2.43271 × 10^−5^
F8	8.352 × 10^−8^	1.20233 × 10^−8^	6.51827 × 10^−9^	1.84999 × 10^−8^	4.07716 × 10^−11^	5.46203 × 10^−6^	1.42942 × 10^−8^	0.325526587	0.000283887
F9	2.15403 × 10^−6^	6.2828 × 10^−6^	4.68563 × 10^−8^	7.38908 × 10^−11^	0.017649028	3.33839 × 10^−11^	4.97517 × 10^−11^	0.001301665	1.25408 × 10^−7^
F10	1.06657 × 10^−7^	3.19674 × 10^−9^	7.38029 × 10^−10^	0.000158461	5.57265 × 10^−10^	0.510597937	1.72941 × 10^−7^	5.57265 × 10^−10^	3.15889 × 10^−10^

**Table 4:** Statistical summary of six performance metrics for ORSBOA vs. comparator algorithms on the CEC2022.

Function	Algorithm	Min	Std	Avg	Median	Worse	Avg_Time
F1	GWO	346.0046	1803.585	1705.042	511.729	6273.765	0.074689
	WOA	3487.383	9724.353	18,600.97	17,307.23	47,115.86	0.081098
	HHO	301.5913	7.250941	308.3948	306.6178	339.9692	0.128083
	BAS	300	1.36 × 10^−5^	300	300	300.0001	0.200927
	COA	2971.339	1870.416	7630.609	7685.623	10,188.76	0.086085
	FOX	300	187.4703	341.4773	300.0001	1307.292	0.078074
	DBO	1515.424	1589.523	4579.583	4563.294	7768.611	0.107462
	OOA	2335.579	2231.095	7615.42	8181.238	10,795.96	0.088272
	SBOA	300	2.87 × 10^−11^	300	300	300	0.39338
	ORSBOA	300	6.38 × 10^−9^	300	300	300	0.095336
F2	GWO	403.5684	24.0486	432.8305	432.7548	470.9885	0.079806
	WOA	401.1798	75.70196	467.6259	457.9632	723.956	0.097359
	HHO	400.0836	30.42944	422.1832	408.9699	512.4899	0.103128
	BAS	400.1553	12.8807	408.9025	407.4695	475.7409	0.165483
	COA	619.6907	481.6783	1570.118	1493.995	2823.828	0.070342
	FOX	400.0013	19.12118	412.3053	405.8008	468.1674	0.058379
	DBO	465.6005	114.3565	574.8781	534.3195	996.0364	0.086123
	OOA	684.3322	897.4724	1761.99	1513.851	3961.34	0.069523
	SBOA	400.0281	32.72611	429.4614	408.9161	475.7097	0.313669
	ORSBOA	400.0193	3.230676	405.7995	405.367	408.9161	0.075346
F3	GWO	600.0375	1.21245	600.9613	600.3638	605.362	0.095403
	WOA	616.7612	14.62737	635.8485	634.3179	669.0912	0.108852
	HHO	605.8137	13.30215	635.5805	636.6164	660.7701	0.183638
	BAS	600.0962	16.88763	620.4436	617.6093	660.1327	0.261403
	COA	627.9786	7.991812	645.4092	645.463	658.2609	0.149529
	FOX	633.279	9.024805	656.083	657.1738	670.8076	0.091066
	DBO	612.9304	5.001888	622.4573	623.9414	629.4338	0.122213
	OOA	625.5393	10.56268	646.3185	647.4845	667.8876	0.151615
	SBOA	600	13.93244	613.6221	609.2783	642.9368	0.377837
	ORSBOA	600	0.133801	600.0244	600	600.7329	0.141794
F4	GWO	806.974	5.50037	814.3812	813.2439	828.8705	0.069826
	WOA	815.3277	13.30663	841.2438	842.3179	867.2701	0.085581
	HHO	811.061	7.661842	827.2159	826.5427	844.9323	0.131585
	BAS	817.9092	14.42068	850.0248	847.9559	881.6581	0.194793
	COA	832.4477	8.398161	850.2692	849.9983	865.6295	0.09479
	FOX	831.8386	6.241407	834.2265	832.8336	861.6871	0.068663
	DBO	818.1685	6.825165	831.7633	830.2759	843.7645	0.094553
	OOA	828.1269	11.03264	846.4656	843.8396	872.1889	0.088106
	SBOA	808.9546	7.314193	829.2517	831.8386	845.7679	0.319798
	ORSBOA	802.9849	3.843106	808.623	807.9597	818.9042	0.095141
F5	GWO	900.1025	7.882356	904.2763	901.0103	934.7091	0.071693
	WOA	1020.832	522.7823	1521.066	1421.883	3249.337	0.087062
	HHO	1099.864	136.2296	1379.301	1388.245	1587.615	0.134328
	BAS	1068.848	834.1822	2178.617	2024.154	4775.017	0.199852
	COA	1068.781	190.2096	1392.016	1418.807	1677.064	0.100387
	FOX	1449.327	68.91695	1481.291	1467.955	1842.824	0.070228
	DBO	953.6449	56.69146	1066.239	1061.136	1257.36	0.097584
	OOA	1156.668	159.7257	1398.848	1394.02	1801.915	0.096345
	SBOA	900.9086	239.5854	1282.361	1424.063	1493.899	0.329545
	ORSBOA	900	0.022714	900.006	900	900.0895	0.103384
F6	GWO	2382.612	2358.473	6088.575	5487.275	9402.936	0.06241
	WOA	2066.429	1511.062	3875.225	3687.162	8114.415	0.078741
	HHO	2009.881	2824.715	5499.185	4973.687	13,636.9	0.110441
	BAS	1846.268	2373.752	4690.372	4862.633	8040.852	0.1693
	COA	1962.6	13,067,983	8,122,081	1,207,475	52,735,978	0.07482
	FOX	1919.619	2218.369	4532.76	4027.768	8199.948	0.060039
	DBO	6828.778	578,434.1	417,805.5	208,703.8	2,357,812	0.088622
	OOA	1855.202	7,823,316	4,030,606	132,387.3	34,941,814	0.074859
	SBOA	1828.116	1829.041	3431.698	2600.003	7707.617	0.325564
	ORSBOA	1910.677	1376.21	3215.867	2993.42	7555.268	0.081043
F7	GWO	2010.834	8.826849	2027.864	2025.109	2054.518	0.100932
	WOA	2032.7	24.76016	2069.825	2062.697	2134.619	0.117424
	HHO	2027.359	25.83524	2069.795	2066.79	2147.864	0.20195
	BAS	2020.978	39.16726	2056.518	2055.989	2159.25	0.288961
	COA	2049.046	20.57153	2081.285	2081.129	2129.376	0.170337
	FOX	2076.343	71.07461	2157.273	2133.693	2331.698	0.09901
	DBO	2030.651	12.65574	2051.857	2050.158	2090.072	0.127218
	OOA	2054.351	23.67721	2090.096	2091.114	2127.993	0.150113
	SBOA	2003.98	43.46529	2052.759	2030.069	2123.143	0.384255
	ORSBOA	2000	9.203731	2007.742	2001.99	2022.614	0.154587
F8	GWO	2203.911	5.265387	2222.943	2223.759	2229.269	0.110257
	WOA	2212.717	8.177929	2234.853	2234.435	2253.444	0.128956
	HHO	2224.624	13.87968	2235.812	2230.908	2269.53	0.251463
	BAS	2219.935	41.72078	2243.821	2222.231	2348.747	0.327455
	COA	2225.343	6.11297	2232.357	2229.966	2248.405	0.202219
	FOX	2228.655	139.708	2427.998	2443.607	2646.627	0.111719
	DBO	2214.736	4.56706	2230.03	2229.783	2237.51	0.140729
	OOA	2225.368	4.293843	2231.449	2230.546	2241.554	0.174095
	SBOA	2200.914	8.031115	2222.388	2221.223	2256.67	0.401379
	ORSBOA	2200.042	9.474163	2212.807	2220.22	2223.177	0.178837
F9	GWO	2529.341	41.60504	2570.246	2549.119	2676.217	0.096796
	WOA	2529.983	47.36422	2588.248	2587.264	2677.367	0.112421
	HHO	2531.293	40.46998	2578.295	2569.118	2676.363	0.189263
	BAS	2529.284	26.82598	2534.182	2529.284	2676.216	0.273115
	COA	2639.762	36.96955	2736.109	2729.919	2842.078	0.163159
	FOX	2529.302	47.23425	2563.17	2544.894	2701.685	0.09585
	DBO	2545.157	51.34999	2634.968	2635.071	2739.702	0.126157
	OOA	2654.202	43.94692	2746.213	2734.408	2886.566	0.142378
	SBOA	2529.284	26.93081	2534.944	2529.284	2676.216	0.428952
	ORSBOA	2529.284	2.07 × 10^−13^	2529.284	2529.284	2529.284	0.18506
F10	GWO	2412.102	122.7353	2576.326	2608.223	3133.79	0.119719
	WOA	2500.601	384.667	2762.013	2636.474	3915.235	0.113258
	HHO	2500.549	80.59077	2587.758	2623.694	2806.774	0.187637
	BAS	2500.826	182.9216	2684.57	2634.872	3088.964	0.273627
	COA	2529.833	256.2705	2838.97	2771.278	3799.068	0.157631
	FOX	2500.65	505.3677	2882.277	2664.331	4021.874	0.092336
	DBO	2501.037	44.59351	2519.293	2505.744	2668.766	0.121526
	OOA	2508.893	140.3412	2699.576	2697.157	3056.034	0.134435
	SBOA	2500.24	0.128525	2500.486	2500.494	2500.833	0.365602
	ORSBOA	2500.24	51.69357	2536.249	2500.354	2611.794	0.142554
F11	GWO	2600.973	154.4511	2960.649	2916.943	3214.997	0.123405
	WOA	2635.601	445.1138	3024.469	2946.267	4662.617	0.142012
	HHO	2605.377	128.8462	2795.938	2750.905	2926.441	0.260146
	BAS	2600	531.6957	3058.189	2900	5163.616	0.356629
	COA	3042.793	462.591	3842.346	3839.079	4599.492	0.227526
	FOX	2600.023	134.9404	2820.047	2900.042	2900.08	0.120918
	DBO	2775.592	111.1419	2866.684	2827.223	3241.167	0.145636
	OOA	2872.029	465.0102	3969.422	4055.25	4562.948	0.196092
	SBOA	2600	142.2588	2710.439	2600	2912.303	0.407314
	ORSBOA	2600	137.2934	2795.043	2900	2900	0.202414
F12	GWO	2858.763	4.670679	2866.314	2865.149	2884.809	0.125578
	WOA	2863.877	27.79651	2884.699	2869.102	2975.045	0.145343
	HHO	2859.57	42.48985	2903.109	2888.729	3033.062	0.263526
	BAS	2862.846	24.47614	2877.154	2868.55	2988.159	0.368845
	COA	2884.75	39.16145	2941.833	2942.476	3005.785	0.234441
	FOX	2892.125	104.8911	3023.91	3007.353	3284.791	0.126109
	DBO	2865.561	15.72066	2881.798	2876.082	2915.344	0.153148
	OOA	2917.325	81.43267	3078.22	3077.82	3235.927	0.197517
	SBOA	2861.405	8.398854	2868.646	2865.166	2903.108	0.41737
	ORSBOA	2858.633	1.996123	2861.92	2862.567	2864.931	0.204626

**Table 5:** Wilcoxon signed-rank test results for ORSBOA vs. comparator algorithms on the CEC2022.

Function	WOA	HHO	BAS	COA	FOX	DBO	OOA	SBOA	ORSBOA
F1	1.7344 × 10^−6^	1.7344 × 10^−6^	1.7344 × 10^−6^	1.7344 × 10^−6^	1.92092 × 10^−6^	3.11232 × 10^−5^	2.35342 × 10^−6^	1.7344 × 10^−6^	1.7344 × 10^−6^
F2	0.035008957	0.059835601	7.51366 × 10^−5^	1.7344 × 10^−6^	0.000962659	1.7344 × 10^−6^	1.7344 × 10^−6^	0.585711569	1.12654 × 10^−5^
F3	1.7344 × 10^−6^	1.7344 × 10^−6^	1.92092 × 10^−6^	1.7344 × 10^−6^	1.7344 × 10^−6^	1.7344 × 10^−6^	1.7344 × 10^−6^	0.000189097	1.7344 × 10^−6^
F4	2.12664 × 10^−6^	2.60333 × 10^−6^	1.7344 × 10^−6^	1.7344 × 10^−6^	1.7344 × 10^−6^	2.35342 × 10^−6^	1.7344 × 10^−6^	6.33914 × 10^−6^	0.00024118
F5	1.7344 × 10^−6^	1.7344 × 10^−6^	1.7344 × 10^−6^	1.7344 × 10^−6^	1.7344 × 10^−6^	1.7344 × 10^−6^	1.7344 × 10^−6^	2.8786 × 10^−6^	1.7344 × 10^−6^
F6	0.000528725	0.416533807	0.040702311	7.69086 × 10^−6^	0.021826722	1.92092 × 10^−6^	4.07151 × 10^−5^	0.0003065	0.000114992
F7	2.8786 × 10^−6^	3.51524 × 10^−6^	0.002414704	1.7344 × 10^−6^	1.7344 × 10^−6^	2.60333 × 10^−6^	1.7344 × 10^−6^	0.075213312	2.35342 × 10^−6^
F8	1.23808 × 10^−5^	2.60333 × 10^−6^	0.042766688	1.92092 × 10^−6^	1.7344 × 10^−6^	3.88218 × 10^−6^	3.88218 × 10^−6^	0.165026566	1.49356 × 10^−5^
F9	0.177907383	0.530440091	1.7344 × 10^−6^	1.7344 × 10^−6^	0.428430029	1.23808 × 10^−5^	1.7344 × 10^−6^	2.37045 × 10^−5^	1.7344 × 10^−6^
F10	0.010444401	0.062682812	0.001483928	2.84342 × 10^−5^	0.020671114	0.082206466	0.000771217	0.006424212	0.027029157
F11	0.845080424	0.000528725	0.191522107	1.7344 × 10^−6^	8.46608 × 10^−6^	0.004681835	1.92092 × 10^−6^	1.7344 × 10^−6^	0.000419551
F12	5.79245 × 10^−5^	5.21649 × 10^−6^	9.71105 × 10^−5^	1.7344 × 10^−6^	1.7344 × 10^−6^	2.84342 × 10^−5^	1.7344 × 10^−6^	0.106394173	6.33914 × 10^−6^

**Table 6:** Wilcoxon rank-sum test results for ORSBOA vs. comparator algorithms on the CEC2022.

Function	WOA	HHO	BAS	COA	FOX	DBO	OOA	SBOA	ORSBOA
F1	6.06576 × 10^−11^	3.01986 × 10^−11^	3.00287 × 10^−11^	1.95678 × 10^−10^	9.7555 × 10^−10^	8.1975 × 10^−7^	5.57265 × 10^−10^	2.36315 × 10^−11^	3.01986 × 10^−11^
F2	0.039167068	0.042066821	8.13446 × 10^−7^	3.01986 × 10^−11^	1.63506 × 10^−5^	3.33839 × 10^−11^	3.01986 × 10^−11^	0.211205674	4.14481 × 10^−8^
F3	3.01986 × 10^−11^	3.01986 × 10^−11^	2.66947 × 10^−9^	3.01986 × 10^−11^	3.01986 × 10^−11^	3.01986 × 10^−11^	3.01986 × 10^−11^	0.001952677	1.59086 × 10^−10^
F4	2.60985 × 10^−10^	3.08105 × 10^−8^	5.4907 × 10^−11^	3.01986 × 10^−11^	3.01986 × 10^−11^	3.4742 × 10^−10^	3.33839 × 10^−11^	7.54964 × 10^−9^	8.83709 × 10^−6^
F5	3.01986 × 10^−11^	3.01986 × 10^−11^	3.01986 × 10^−11^	3.01986 × 10^−11^	3.01986 × 10^−11^	3.01986 × 10^−11^	3.01986 × 10^−11^	1.85673 × 10^−9^	2.84303 × 10^−11^
F6	0.000300589	0.245813802	0.004225918	1.38525 × 10^−6^	0.004427193	1.20567 × 10^−10^	0.000526404	1.09069 × 10^−5^	1.86085 × 10^−6^
F7	1.46431 × 10^−10^	4.61591 × 10^−10^	0.012732115	3.68973 × 10^−11^	3.01986 × 10^−11^	3.82489 × 10^−9^	3.33839 × 10^−11^	0.217016825	4.1825 × 10^−9^
F8	6.51827 × 10^−9^	4.1825 × 10^−9^	0.911708975	1.69472 × 10^−9^	3.68973 × 10^−11^	6.01039 × 10^−8^	2.0338 × 10^−9^	0.001679756	1.35943 × 10^−7^
F9	0.099257608	0.290472149	5.06395 × 10^−10^	3.68973 × 10^−11^	0.304176818	5.09117 × 10^−6^	3.68973 × 10^−11^	8.06104 × 10^−9^	6.31878 × 10^−12^
F10	0.000283887	0.001442328	1.74791 × 10^−5^	3.01026 × 10^−7^	0.000556111	0.830255284	2.59736 × 10^−5^	0.087710377	0.00185748
F11	0.773119942	0.004032978	0.001380325	1.46431 × 10^−10^	9.06321 × 10^−8^	0.001003532	9.7555 × 10^−10^	3.093 × 10^−9^	4.88162 × 10^−8^
F12	8.29194 × 10^−6^	1.06657 × 10^−7^	0.000124771	3.33839 × 10^−11^	3.01986 × 10^−11^	7.08811 × 10^−8^	3.01986 × 10^−11^	0.455296907	3.5148 × 10^−7^

**Table 7:** Statistical summary for the welded beam design problem: ORSBOA vs. comparator algorithms.

Algorithm	Min	Std	Avg	Median	Worse	Avg_Time	Wilcoxon Signed-Rank Test	Wilcoxon Rank-Sum Test
GWO	1.671519	0.002541	1.675055	1.674698	1.680911	0.115776		
WOA	1.742417	1.020821	2.49263	2.025903	4.879656	0.138523	6 × 10^−100^	1 × 10^−186^
HHO	1.755779	0.224695	1.950468	1.80961	2.365512	0.28435	4.1 × 10^−99^	9.6 × 10^−183^
BAS	1.70743	0.128668	1.803455	1.74935	2.124741	0.379334	1.2 × 10^−79^	1.67 × 10^−81^
COA	1.901363	0.121738	2.091732	2.120144	2.201244	0.264057	2.72 × 10^−96^	2.6 × 10^−178^
FOX	1.819554	0.17747	2.060101	2.064732	2.329194	0.139037	1.33 × 10^−98^	2.1 × 10^−179^
DBO	1.69453	0.071151	1.780323	1.784546	1.905103	0.164816	1.18 × 10^−98^	2.7 × 10^−166^
OOA	2.924624	0.415338	3.458877	3.391045	4.07709	0.219622	6 × 10^−100^	3.9 × 10^−191^
SBOA	1.670218	0.250451	1.926727	1.859669	2.409164	0.392814	8.81 × 10^−97^	1.2 × 10^−164^
ORSBOA	1.670218	1.17 × 10^−5^	1.670225	1.670221	1.670254	0.227155	2.69 × 10^−75^	2.1 × 10^−121^

**Table 8:** Statistical summary for the three-bar truss design problem: ORSBOA vs. comparator algorithms.

Algorithm	Min	Std	Avg	Median	Worse	Avg_Time	Wilcoxon Signed-Rank Test	Wilcoxon Rank-Sum Test
GWO	263.8981	0.007558	263.9062	263.9038	263.9199	0.10394		
WOA	263.9267	4.2413	266.0873	264.2168	277.5173	0.12383	5.9 × 10^−100^	8.4 × 10^−185^
HHO	263.8972	0.192497	264.0701	264.0202	264.4576	0.261431	5.89 × 10^−97^	3.7 × 10^−126^
BAS	263.8958	0.019939	263.909	263.8967	263.9552	0.31928	3.35 × 10^−80^	3.1 × 10^−110^
COA	263.896	0.022494	263.9054	263.8985	263.9691	0.228751	1.5 × 10^−99^	9.3 × 10^−137^
FOX	263.896	0.002554	263.8973	263.8966	263.9044	0.122567	5.9 × 10^−100^	4.32 × 10^−96^
DBO	263.9172	0.334727	264.1298	263.9649	264.8302	0.136174	1.79 × 10^−97^	5.3 × 10^−165^
OOA	264.1219	2.155156	266.3746	266.0914	270.2686	0.205727	5.9 × 10^−100^	1.7 × 10^−185^
SBOA	263.8959	0.001018	263.8969	263.8967	263.8986	0.373537	6 × 10−100	1.7 × 10^−127^
ORSBOA	263.8958	0.000152	263.8959	263.8959	263.8963	0.204208	1.09 × 10^−98^	5.8 × 10^−156^

**Table 9:** Statistical summary for the cantilever beam design problem: ORSBOA vs. comparator algorithms.

Algorithm	Min	Std	Avg	Median	Worse	Avg_time	Wilcoxon Signed-Rank Test	Wilcoxon Rank-Sum Test
GWO	1.340008	6.73 × 10^−5^	1.340096	1.340094	1.340248	0.040781		
WOA	1.35682	0.341661	1.586548	1.500634	2.527584	0.056036	3.86 × 10^−95^	5.6 × 10^−179^
HHO	1.341229	0.003691	1.345286	1.344313	1.353627	0.08054	1.07 × 10^−83^	1.09 × 10^−33^
BAS	1.339986	0.000405	1.340338	1.340239	1.341309	0.122796	1.18 × 10^−84^	1.2 × 10^−137^
COA	1.369427	0.063574	1.444664	1.449096	1.541185	0.069015	9.88 × 10^−80^	1 × 10^−166^
FOX	1.339959	2.67 × 10^−5^	1.339991	1.339989	1.340036	0.04637	1.97 × 10^−58^	7.89 × 10^−12^
DBO	1.340111	0.000283	1.340357	1.340236	1.340992	0.062602	4.18 × 10^−96^	1.06 × 10^−41^
OOA	1.496922	0.024753	1.528269	1.522125	1.56011	0.063982	7.3 × 10^−85^	7.5 × 10^−174^
SBOA	1.339957	5.83 × 10^−5^	1.340011	1.339999	1.340157	0.216642	6 × 10^−100^	5.1 × 10^−139^
ORSBOA	1.339957	3.51 × 10^−6^	1.339962	1.339961	1.339968	0.069479	1.58 × 10^−61^	2.5 × 10^−120^

**Table 10:** Statistical summary for the compression spring design problem: ORSBOA vs. comparator algorithms.

Algorithm	Min	Std	Avg	Median	Worse	Avg_Time	Wilcoxon Signed-Rank Test	Wilcoxon Rank-Sum Test
GWO	0.01267	9.55 × 10^−5^	0.012767	0.012736	0.013018	0.118662		
WOA	0.012813	0.001144	0.014062	0.013848	0.016002	0.14385	9.31 × 10^−29^	7.86 × 10^−61^
HHO	0.012668	0.001193	0.013553	0.012982	0.01609	0.279993	2.12 × 10^−6^	5.91 × 10^−33^
BAS	0.012676	0.001582	0.013581	0.012737	0.01777	0.367191	1.31 × 10^−21^	1.74 × 10^−37^
COA	0.012725	0.002732	0.013893	0.012849	0.021509	0.283145	2.4 × 10^−88^	9.36 × 10^−77^
FOX	0.012677	0.000431	0.012961	0.012732	0.013841	0.131778	2.31 × 10^−97^	3.29 × 10^−26^
DBO	0.012876	0.002332	0.014935	0.013415	0.01814	0.161107	1.17 × 10^−98^	1.2 × 10^−173^
OOA	0.012826	2.15 × 10^14^	1.63 × 10^14^	4.84 × 10^13^	6.32 × 10^14^	0.227287	6.7 × 10^−102^	2.6 × 10^−196^
SBOA	0.01271	0.000206	0.012954	0.012962	0.013326	0.394917	5.94 × 10^−55^	4.12 × 10^−26^
ORSBOA	0.012666	2.09 × 10^−5^	0.012695	0.012691	0.012729	0.229789	8.06 × 10^−95^	7.5 × 10^−133^

## Data Availability

The data that support the findings of this study are available from the corresponding author upon request. There are no restrictions on data availability.
